# Maternal docosahexaenoic acid supplementation during lactation improves exercise performance, enhances intestinal glucose absorption and modulates gut microbiota in weaning offspring mice

**DOI:** 10.3389/fnut.2024.1423576

**Published:** 2024-07-05

**Authors:** Dalu Lu, Die Yao, Gaoli Hu, Jiefei Zhou, Xiuhua Shen, Linxi Qian

**Affiliations:** ^1^Shanghai Key Laboratory of Pediatric Gastroenterology and Nutrition, Xinhua Hospital, Shanghai Jiao Tong University School of Medicine, Shanghai, China; ^2^Shanghai Institute for Pediatric Research, Xinhua Hospital, Shanghai Jiao Tong University School of Medicine, Shanghai, China; ^3^Department of Clinical Nutrition, College of Health Science and Technology, Shanghai Jiao Tong University School of Medicine, Shanghai, China; ^4^School of Public Health, Shanghai Jiao Tong University School of Medicine, Shanghai, China

**Keywords:** docosahexaenoic acid, lactation, exercise, glucose absorption, microbiota, mTOR pathway

## Abstract

**Introduction:**

Intestinal dysfunction induced by weaning stress is common during breastfeeding period. Docosahexaenoic acid (DHA) is well known for promoting visual and brain development, but its effects on early intestinal development remain unknown. This study investigated the impact of maternal DHA supplementation during lactation on intestinal glucose absorption and gut microbiota in weaning offspring mice.

**Materials and methods:**

Dams were supplemented with vehicle (control), 150 mg/(kg body weight · day) DHA (L-DHA), or 450 mg/(kg body weight · day) DHA (H-DHA) throughout lactation by oral administration. After weaning, pups were randomly divided into three groups for athletic analysis, microbial and proteomic analysis, biochemical analysis, 4-deoxy-4-fluoro-D-glucose (4-FDG) absorption test, and gene expression quantitation of glucose transport-associated proteins and mTOR signaling components.

**Results:**

The H-DHA group exhibited enhanced grip strength and prolonged swimming duration compared to the control group. Additionally, there were significant increases in jejunal and ileal villus height, and expanded surface area of jejunal villi in the H-DHA group. Microbial analyses revealed that maternal DHA intake increased the abundance of beneficial gut bacteria and promoted metabolic pathways linked to carbohydrate and energy metabolism. Proteomic studies indicated an increased abundance of nutrient transport proteins and enrichment of pathways involved in absorption and digestion in the H-DHA group. This group also showed higher concentrations of glucose in the jejunum and ileum, as well as elevated glycogen levels in the liver and muscles, in contrast to lower glucose levels in the intestinal contents and feces compared to the control group. The 4-FDG absorption test showed more efficient absorption after oral 4-FDG gavage in the H-DHA group. Moreover, the expressions of glucose transport-associated proteins, GLUT2 and SGLT1, and the activation of mTOR pathway were enhanced in the H-DHA group compared to the control group. The L-DHA group also showed similar but less pronounced improvements in these aspects relative to the H-DHA group.

**Conclusion:**

Our findings suggested that maternal DHA supplementation during lactation improves the exercise performance, enhances the intestinal glucose absorption by increasing the expressions of glucose transporters, and beneficially alters the structure of gut microbiome in weaning offspring mice.

## Graphical abstract

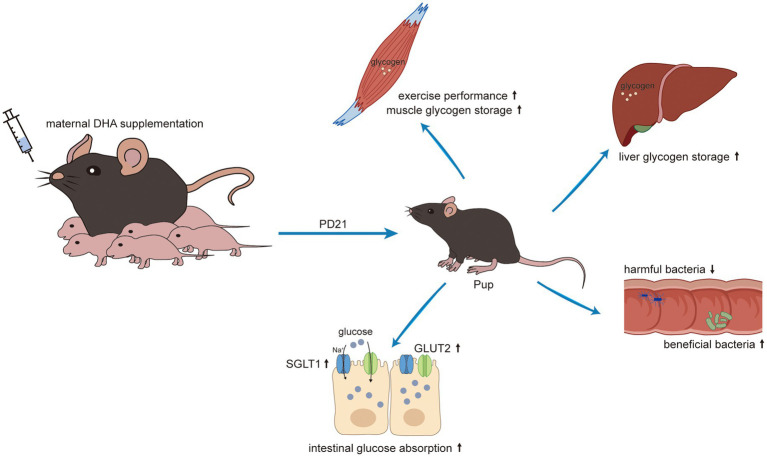

## 1 Introduction

Nutrition during the prenatal and infancy stages is considered to be a critical (non-genetic) determinant for growth and development. Breastfeeding recommended by the World Health Organization (WHO) is considered as the best feeding modality for infants (1, 2). It is reported that breast milk composition is closely related to maternal dietary intake (3). Therefore, maternal nutrition during pregnancy and lactation has a momentous impact on the long-term health of offspring. As the ideal nutritional source for infants, especially for the first 6 months of life, breast milk contains a multitude of basic nutrients and bioactive ingredients that are essential for the maturation of the gut in infants, such as immunoglobulin, oligosaccharides, short-chain fatty acids, antimicrobial peptides, and microbes. Moreover, a previous research suggested that the extent of breastmilk’s benefits is affected by its fatty acid composition (4). Fatty acids are essential components of breast milk, which provide structure and modulate functions of cell membranes, synthesize signaling molecules as cellular messengers in signal-transduction pathways, and mediate and regulate immune functions (5). Docosahexaenoic acid (DHA), a prominent n-3 long-chain polyunsaturated fatty acids (LCPUFAs) in human breast milk, has been identified to play a central role in the development of the infant visual and brain neural systems and is also known to be involved in membrane fluidity, neurogenesis, synaptogenesis, anti-inflammatory and anti-oxidative activities (6). Although it is possible to convert α-linolenic acid to DHA by elongase and desaturase enzymes in the body, studies have shown that only small amounts of DHA can be synthesized by this process (7). Thus, maternal supplementation with no less than 200 mg of DHA per day is recommended during lactation to meet the requirements both of the mother and the suckling infant (8).

Early life is a vital period for the development of the gut microbiota and the intestinal physical barrier and digestive functions, and the maturation and health of the gut in this period have been found to be associated with programming health and disease later in adulthood (9). In the early stages of rapid development, intestinal maturation in infancy relies heavily on adequate nutritional support from breast milk for optimal development of digestive system. As the immaturity of the gastrointestinal tract and changes in food composition, post-weaning disturbed digestive function is one of the important problems in infant feeding (10). A growing body of evidence has shown that the weaning period, a critical transition period for mammalian neonates, becomes more susceptible to be accompanied by intestinal bacterial disorders and inflammatory reactions. Prolonged intestinal inflammation caused by pathogens can damage the intestinal epithelium and compromise the nutrient digestion and absorption, which leads to growth retardation and malnutrition in subsequent early childhood (11, 12). The early-life gut microbiota of offspring is primarily shaped by breast milk, which is known as the entero-mammary axis (13). During weaning, the gut microbiota shifts to a new stage of development and evolves towards a composition similar to that of adult individuals due to the introduction of solid food. A stable gut microbiota system can help neonates adapt to such transition and reduce the incidence of dysbiosis. Accordingly, it is necessary to promote the intestinal development and maintain the gut homeostasis of infants to optimize their performance during this critical stage. Previous studies have reported that maternal supplementation of fish oil, a complex consisting mainly of DHA, during pregnancy and lactation significantly facilitates the suckling-to-weaning transition by altering the gut microbial community and improves intestinal nutrient uptake and energy stores in weaning piglets (14, 15). These findings suggested that DHA may positively affect gut development in early life.

To date, trials that have explored the effect of early DHA supplementation have been mainly focused on brain development and cognitive assessments, but studies on the effects of DHA on intestinal nutrient absorption functions are limited. It is a cost-effective intervention that provides a specific maternal diet to promote the intestinal development of offspring. Consequently, on the basis of previously published research, we further investigated the potential influence of maternal DHA supplementation during lactation on intestinal glucose absorption capacity and gut microbiota colonization of weaning offspring using mice as a model. In addition, we also explored whether such supplementation could enhance tissue glycogen stores and improve exercise performance in offspring.

## 2 Materials and methods

### 2.1 Animals and experimental design

Pregnant female C57BL/6 mice at gestational day (GD)16 were purchased from Shanghai Jihui Laboratory Animals (China). All animals were housed in ventilated cages with a 12 h light/dark cycle at a constant temperature (21–23°C) and were provided with food and water *ad libitum*. The compositions of rodent chow diet were provided in Supplementary Table S1. The fatty acid compositions of this chow diet were provided in Supplementary Table S2. C57BL/6 dams were randomly assigned into three groups (*n* = 6): received without DHA supplementation orally (control group), received with 150 mg/(kg body weight · day) DHA supplementation orally (L-DHA group), and received with 450 mg/(kg body weight · day) DHA supplementation orally (H-DHA group). DHA (purity >98%) was purchased from Macklin (China). We dissolved DHA in ddH_2_O containing 0.5% CMC-Na (Solarbio, China) to obtain 15 mg/mL and 45 mg/mL DHA solutions. From GD18 to postpartum day (PD) 21, DHA solutions at low and high concentrations were administrated to the dams from L-DHA and H-DHA groups, respectively, by oral gavage daily, while the dams from the control group received an equal volume of vehicle daily. The DHA doses in this study were based on amounts used in previous clinical trials with humans and adjusted for the 12-fold higher metabolic rate in mice (16–19). Accordingly, the doses of 150 and 450 mg/kg body weight per day for mice correspond to approximately 750 and 2,250 mg per day for humans, respectively. After delivery, pups were raised by their own mothers from PD0 to PD21. To guarantee offspring homogeneity, each litter was adjusted to 6 pups with equal numbers of males and females wherever possible. The body weight of pups was recorded every day from PD7 to PD21.

**Figure 1:**
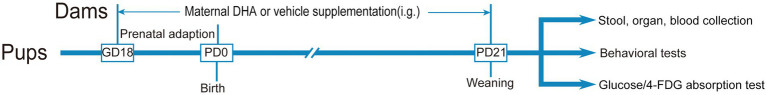
A scheme of the experimental design.

At weaning (PD21), three random selections with one male and one female pup per litter for each selection were carried out. The random sampling method was used in each selection. The pups from the first selection were used for sample collection (*n* = 12). Briefly, fresh stool feces from each pup were first collected after overnight fasting and stored at −80°C. Immediately after feces collection, offspring were sacrificed under anesthesia with sodium pentobarbital (45 mg · kg^−1^, i.p.) and autopsied. Organs were carefully excised, rinsed in saline solution and blotted dry. The weight of the brain, heart, liver, spleen, kidney and the length of the small intestine were recorded for growth and developmental assessment. The blood samples and the contents in ileum and colon were also collected. Serum was separated from whole blood samples by centrifugation at 5000 rpm for 10 min at 4°C and stored at −80°C. Tissues of liver, gastrocnemius muscle, jejunum, ileum, colon and the contents in ileum and colon canal were collected and stored at −80°C. Part of jejunal and ileal segments was immersed in 4% paraformaldehyde for histochemical staining. The pups from the second selection were used for forelimb grip strength test and weight-loaded swimming test (*n* = 12), while the pups from the third selection were used for glucose/4-deoxy-4-fluoro-D-glucose (4-FDG) absorption test (*n* = 8). A schematic of the experimental design was shown in Figure 1.

### 2.2 Determination of glycogen content in liver and muscle and glucose content in intestine, intestinal contents and feces

The glycogen level in liver and muscle tissue was determined using the glycogen assay kit (Nanjing Jiancheng, China). The glucose level in jejunum, ileum, colon tissue, and contents in ileum and colon canal, and feces was determined using the glucose assay kit (Nanjing Jiancheng). The final concentration was normalized to the initial wet weight of the specimen.

### 2.3 Examination of serum biochemical parameters

Total serum cholesterol (TC), triglyceride (TG), protein (TP) and serum glucose were estimated by commercial assay kits (Nanjing Jiancheng). All procedures were conducted according to the manufacturer’s instructions.

### 2.4 Fatty acid analysis

Fifty milligram of chow diet and jejunum tissue were crushed into fine powder in liquid nitrogen, and total lipids were extracted using chloroform:methanol (2:1). Lipid samples were then saponified with 0.5 M methanolic NaOH for 5 min in boiling water. The hydrolyzed sample was cooled and added boron triflouride solution in methanol (10%). The solution was heated in boiling water bath for 30 min. Extraction was carried out by adding n-hexane and vortex-mixing for 30 s. After centrifugation, the supernatant was collected, and methyl nonadecanoate (C19:0) was added to the extract as an internal standard. Fatty acid methyl esters in the upper hexane layer were analyzed by GCMS-QP2010 Ultra instrument (SHIMADZU, Japan) equipped with a SP-2560 capillary column (100 m × 0.25 mm × 0.2 μm, Sigma-Aldrich, USA). Determination of the fatty acid methyl esters was performed using standard spectrogram from the NIST Mass Spectral Database. Quantitation was determined after normalization to the internal standard. Detailed procedures of fatty acid analysis can be seen in Ochin’s study (20).

### 2.5 Small intestine morphology

After fixation, jejunum and ileum samples were embedded in paraffin and cut into 5-μm-thick sections for hematoxylin and eosin (H&E) staining. The stained sections were observed under light microscopy (Eclipse 80i, Nikon, Japan). The villus height, the villus width and the crypt depth of ten vertically well-orientated villi and crypts were determined using an analysis system (NIS-elements suite; Nikon) by experienced observers who were unaware of treatment group (D.L. and G.H.). The villus height was determined from the crypt opening to the top of the villus and crypt depth from the base of the crypt to its opening. The villus width was defined as the distance from the outside epithelial edge to the outside of the opposite epithelial edge at the half height of the villus. The ratio of villus height to crypt depth (V/C) was calculated to indicate the maturity of villus. The villi surface area was calculated from the villus height and the villus width according to Kolba et al. (21).

### 2.6 Forelimb grip strength test

The detailed procedure of forelimb grip strength test was performed as described in the previous publication (22). Briefly, a grip strength meter adapted from a digital tension apparatus (SF-3, AIPLI, China) was used to measure the forelimb grip strength of offspring after weaning at PD21. Each pup was allowed to grasp the grid of the device with both forepaws and its tail was gently pulled back until both forepaws released the grid. The maximum pull force was recorded by the tension apparatus. Measurements were discarded when mice only used one paw, used its hind paws, turned backwards during the pull, or released the bar without resistance. Each pup had five times to perform the test with a 5-min rest between each test. The values, excluding the highest and lowest results, were normalized to the body weight of each pup.

### 2.7 Weight-loaded swimming test

The weight-loaded swimming test was performed 1 h after the forelimb grip strength test, as described previously in the previous publication (23). Each mouse, which was loaded with a lead block weighing 5% of the body weight on its tail, was placed individually in a plastic pool (50 × 40 × 40 cm) filled with water (25 ± 1°C). The pool water was kept flowing by gently stirring with a glass rod, allowing the mice to swim to exhaustion during the swimming test. The weight-loaded swimming time, used as an index of exercise endurance, was recorded when the mice sank into the water and failed to rise to the surface for breath within 10 s. After the test, the mouse was removed from the water and dried immediately.

### 2.8 Glucose and 4-FDG absorption test

At weaning (PD21), after fasting overnight, 2 g/kg glucose and 10 mg/kg 4-FDG (a glucose analog) were orally administered together followed by taking blood samples from the tail vein at 0, 15, 30, 60 and 120 min and from the mandibular vein at 30 and 60 min. Glucose levels were measured using a glucometer (Bayer Healthcare LLC, Germany). The blood glucose changes over time (0, 15, 30, 60 and 120 min) in response to oral glucose gavage were used to indicate the function of intestinal glucose absorption and overall glucose tolerance. The area under the curve (AUC) of the glucose response curve was calculated to indicate the overall glucose tolerance. The changes of blood 4-FDG concentration in response to oral 4-FDG gavage were used to indicate the function of intestinal glucose absorption. Serum obtained from blood collected at 30 and 60 min was mixed with the same volume of HPLC buffer (55% 100 mM potassium phosphate, pH 7, and 45% methanol), and then analyzed by HPLC for 4-FDG quantification (24).

### 2.9 Microbial 16S rRNA sequencing

The stool samples in all groups were sent to BGI Genomics (China) for 16S rRNA gene sequencing. Bacterial DNA was extracted from the fecal contents following the manufacturer’s protocol (DNeasy PowerSoil Kit; Qiagen, Germany). The quality and quantity of DNA were verified by agarose gel electrophoresis and NanoDrop. The DNA extracted from all samples was stored at −20°C until sequencing. The extracted DNA was used as a template for the PCR amplification of bacterial 16S rRNA genes with the barcoded primers. The V3-V4 variable regions of the 16S rRNA genes were amplified using the universal primers 338F (5’-ACTCCTACGGGAGGCAGCAG-3′) and 806R (5’-GGACTACHVGGGTWTCTAAT-3′). The amplicon quality was visualized using gel electrophoresis, purified using the QIAquick Gel Extraction kit (Qiagen), and quantified using the Qubit dsDNA HS Assay Kit (ThermoFisher, United States) according to the manufacturer’s protocols. Equal amounts of purified amplicons were pooled for sequencing using the MGISEQ-2000 platform PE300 (BGI Genomics).

### 2.10 Protein preparation and LC–MS/MS experiments

Lysis buffer (8 M urea, 4% CHAPS, 40 mM Tris, 65 mM DTT) was added into the jejunum tissue homogenized in liquid nitrogen for protein extraction, and sonicated at 20% amplitude for the total working time of 2 min with 5 s on and 5 s off (JY92-IIDN, Scientz Biotechnology, China). The proteins were denatured at 95°C for 5 min. Lysates were centrifuged at 12,000 rpm for 10 min to remove the insoluble debris and retain the supernatant for proteomic experiment. The protein concentration of the supernatant was determined using the tryptophan-based fluorescence quantification method (21586754). Equal amounts of protein samples were digested by filter-aided sample preparation (FASP) procedure with 10 kDa centrifugal filter tubes (Millipore, United States) and centrifuged at 12,000 g at 22°C. First, the protein was washed with urea buffer (8 M urea in 0.1 M Tris–HCl, pH 8.5) three times, and incubated with 50 mM iodoacetamide for 30 min in the darkness. Then, the iodoacetamide was removed by centrifugation. Continually, the protein was washed with urea buffer three times and then washed thrice with 50 mM ammonium bicarbonate. Next, proteins were digested with Trypsin (Promega, United States) in 50 mM ABC at a concentration of 1:50 (w/w) at 37°C for 18 h. Digested peptides were collected at 12,000 g centrifugation. Concentration of eluted peptides was determined by bicinchoninic acid method. 400 μg peptides were dried by vacuum freeze dryer and resolved in 0.1% formic acid. All the prepared samples were analyzed on a LC–MS platform (Bruker Daltonics, United States) consisting of a nanoElute UHPLC coupled to a timsTOF Pro mass spectrometer, and data acquisition was performed in the data independent acquisition (DIA) mode.

### 2.11 Quantitative real-time PCR

Total RNA from jejunum tissue was extracted using Trizol reagent (RNAiso Plus, Takara, Japan), following manufacturer’s instructions. RT reaction (10 μL) was carried out using the Prime Script RT Reagent Kit (Takara). Two-step reverse transcription-quantitative PCR reactions were performed in a qPCR system (QuantStudio Dx, Applied Biosystems) using SYBR GREEN Master Mix reagent kits (Yeasen, China). The primer sequences for glucose transporter 2 (*GLUT2*), sodium glucose co-transporter 1 (*SGLT1*), peptide transporter 1 (*PEPT1*), alanine-serine-cysteine transporter 2 (*ASCT2*), sodium-dependent neutral amino acid transporter 1 (*B0AT1*), excitatory amino acid transporter 1 (*EAAT1*), excitatory amino acid transporter 3 (*EAAT3*), L-type amino acid transporter 1 (*y^+^LAT1*), 4F2 cell-surface antigen heavy chain (*4F2hc*), cationic amino acid transporter 1 (*CAT1*), Niemann Pick C1 like 1 (*NPC1L1*), apolipoprotein A-1 (*APOA1*), apolipoprotein A-4 (*APOA4*), apolipoprotein B (*APOB*), *CD36*, fatty acid binding protein 2 (*FABP2*), fatty acid binding protein 4 (*FATP4*) and *β-actin*, synthesized by Sangon Biotech (China), were provided in Supplementary Table S3. The housekeeping gene β-actin was used for normalization. qPCR results were analyzed using the 2 − ΔΔCt method.

### 2.12 Western blot analysis

Total protein was isolated from jejunum tissues and then lysed using radioimmunoprecipitation assay (RIPA) buffer (Beyotime Biotech, China) supplemented with a 1% PMSF (Beyotime Biotech) and 1% phosphatase inhibitor mixture (Servicebio, China) on ice. Total protein was quantitated using BCA assay kit (Beyotime Biotech). Aliquots (50 μg total proteins) were loaded into SDS/polyacrylamide gels and transferred on to a PVDF-Plus membrane (Millipore). After transfer, the PVDF membrane was blocked for 1 h in 5% fat-free milk and then was, respectively, incubated overnight at 4°C with dilutions of primary antibodies: GLUT2 (A12307, 1:2000, Abclonal, China), SGLT1 (bs-1128R, 1:2000, Bioss, China), S6 ribosomal protein (abs131865, 1:2000, Absin, China), p-Ser240/244-S6 ribosomal protein (ab215214, 1:2000, Abcam, UK), p70S6 kinase (14485-1-AP, 1,1,000, Proteintech, China), p-Thr389-p70S6 kinase (9,234, 1,1,000, Cell Signaling Technology, United States), mammalian target of rapamycin (mTOR, T55306, 1:2000, Abmart, China), p-S2448-mTOR (5,536, 1,1,000, Cell Signaling Technology), β-actin (T40104, 1:4000, Abmart). The bands were detected with horseradish peroxidase-conjugated goat anti-rabbit or anti-mouse IgG secondary antibody (Beyotime Biotech), followed by the use of a chemiluminescence system (Pierce, United States) and ChemiDOC XRS+ imaging system (Bio-Rad, United States). Protein expression was normalized to β-actin, whereas phosphorylated protein was normalized to the total target protein. Band intensities were quantified with Image Lab (Bio-Rad).

### 2.13 Bioinformatic analysis

16S rRNA sequencing data were preprocessed to detect and remove ambiguous bases and low-quality sequences using Trimmomatic software. After trimming, FLASH software was used in the assembly of paired-end reads. Sequences were further denoised using QIIME software, and clustered to generate operational taxonomic units (OTUs) using USEARCH software. The representative read of each OTU was selected using QIIME, and blasted against the RDP database (Release 16). The estimators of α-diversity, including community richness (Chao index) and diversity (Simpson and Shannon index), were calculated using Mothur software. The β-diversity analysis, including principal coordinates analysis (PCoA) and unweighted pair group method with arithmetic mean (UPGMA), was performed using QIIME. Differences of microbiota from phylum down to genus level among groups were discovered using linear discriminant analysis coupled with effect size measurements (LEfSe), and compared using Kruskal–Wallis test. The functional features of 16S rRNA sequencing data was predicted using PICRUST2 software. The predicted functional profiles were then collapsed into KEGG (Kyoto Encyclopedia of Genes and Genomes) database pathways and the differences between the groups were compared by Kruskal-Wallis test.

Protein identification and quantification were conducted using the library-free search function in DIA-NN with the UniProt mouse reference proteome (release version 2019/10). Proteins identified in more than 50% of the samples in each group were retained for further analysis. Missing values were imputed based on the k-nearest neighbor method. Protein intensity was calculated by summing the intensity of their respective peptides. Student’s *t* test was used to compare the differences between two groups by R language. Fold Change (FC) > 1.5 or < 0.67 and *p* < 0.05 were set as the criteria for significant changes between any of the two groups. Bioinformatic analyses including PCoA, Kernel density plot analysis, Pearson correlation coefficient analysis, volcano plot analysis, Gene Ontology (GO) annotation enrichment analysis, and KEGG annotation enrichment analysis were conducted by R language.

### 2.14 Statistical analysis

All data were presented as the mean ± standard error of the mean (SEM). Two-way repeated-measures analysis of variance (ANOVA) was performed to compare the body weight, blood glucose and 4-FDG levels of glucose/ 4-FDG absorption test at various time points among multiple groups. The differences in other indices among groups were analyzed using Student’s t-test when two groups were compared, or one-way ANOVA when multiple groups were compared. Tukey’s test was used to assess the statistical significance between groups following ANOVA tests. Correlation analyses between the relative abundance of bacterial taxa and the relative mRNA or protein expression of glucose transporters were performed using the Spearman correlation coefficient test. *p* value <0.05 was considered to be statistically significant. GraphPad Prism 8.0 was used for statistical analysis.

## 3 Results

### 3.1 Maternal DHA supplementation during lactation has no effect on body weight and organ weight of offspring mice

**Figure 2:**
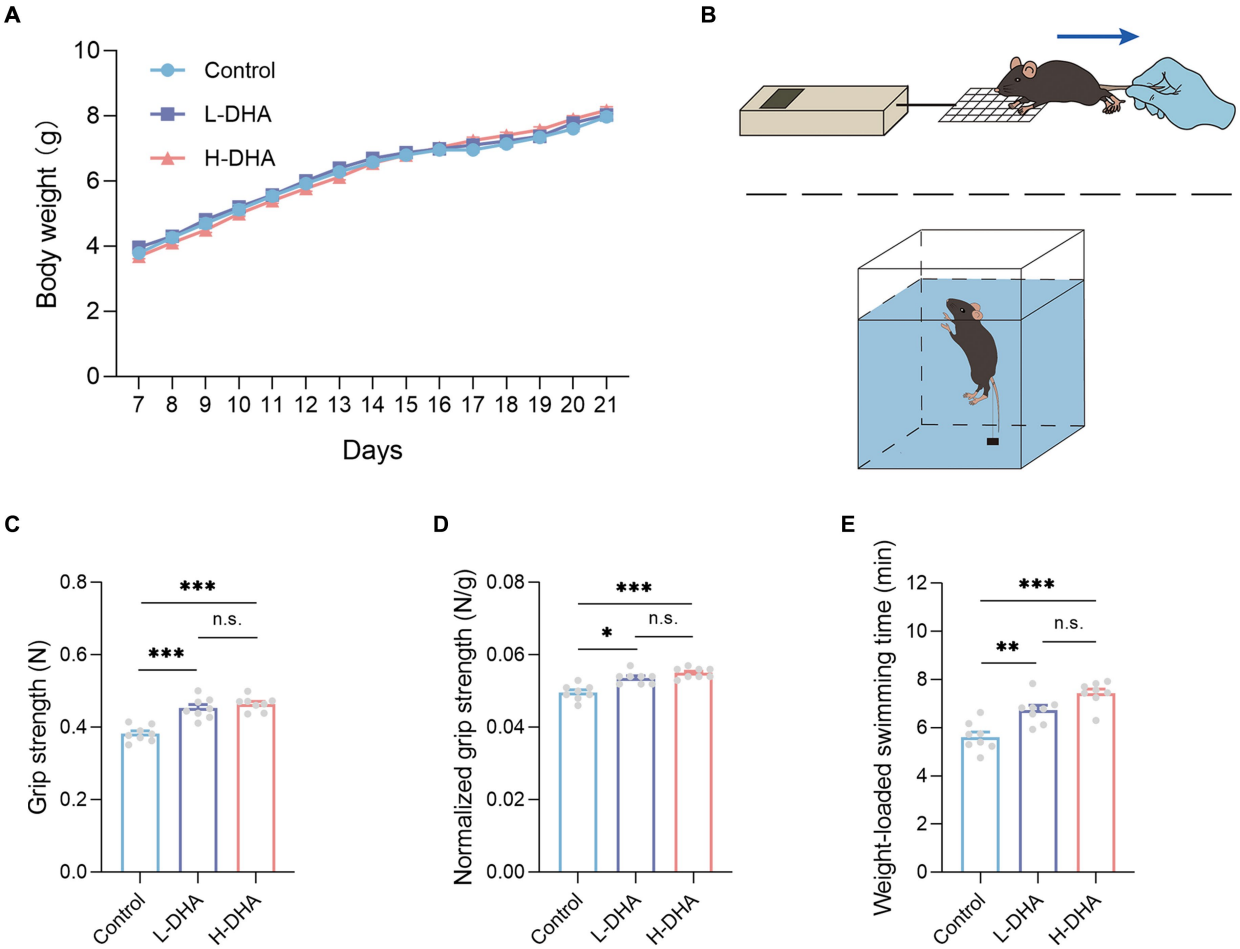
Effect of maternal DHA supplementation during lactation on the body weight and the exercise performance in weaning mice. **(A)** Growth curves of mice from different groups during suckling period. *n* = 12 per group. **(B)** Schematics of the grip strength test and the weight-loaded swimming test. **(C)** Forelimb grip strength and **(D)** normalized forelimb grip strength of mice from different groups. *n* = 8 per group. **(E)** Weight-loaded swimming time of mice from different groups. *n* = 8 per group. Data were expressed as the mean ± SEM. Significance was set at *p* < 0.05 (^*^p < 0.05, ^**^p < 0.01, ^***^p < 0.001). L-DHA: 150 mg/(kg body weight day) DHA; H-DHA: 450 mg/(kg body weight · day) DHA. DHA, docosahexaenoic acid.

To explore the possible effect of maternal DHA supplementation during lactation on body weight and organ weight of pups during the suckling period, we monitored the body weight every day from PD7 to PD21, and recorded organ weight and calculated relative organ weight at the end of the study. However, as shown in Figure 2A, no statistical difference in body weight by 2-way ANOVA analysis was observed among the three groups. The organ weight and relative organ weight also did not differ among the three groups (Supplementary Table S4).

### 3.2 Maternal DHA supplementation during lactation improves exercise performance of offspring mice

As the diagram shown in Figures 2B, the two physical performance tests, including forelimb grip strength and weight-loaded swimming, were conducted. In the forelimb grip strength test, we observed a significant increase in the L-DHA group (18% increase) and the H-DHA group (21% increase) compared with the control group (Figure 2C; *p* < 0.001 and *p* < 0.001, respectively). Meanwhile, relative grip strength (%), calculated by normalization to individual body weight, was 7 and 11% greater in the L-DHA group and the H-DHA group than in the control group (Figure 2D; *p* < 0.05 and *p* < 0.001, respectively). In addition, compared to the control group, the L-DHA group (20% increase) and the H-DHA group (32% increase) also performed longer swimming time, and the trend was significant (Figure 2E; both *p* < 0.01). Whereas, no significant difference of grip strength, normalized grip strength and weight-loaded swimming time was found between the L-DHA group and the H-DHA group.

### 3.3 Effects of maternal DHA supplementation during lactation on serum biochemical indexes of offspring mice

To determine the nutritional effects of maternal DHA supplementation, the serum glucose, TP, TC and TG of pups were examined. The serum biochemical parameters were summarized in Table 1. Markedly, the serum TG concentration was 29 and 36% higher in the L-DHA group and the H-DHA group than that in the control group (*p* < 0.05 and *p* < 0.01, respectively). However, there was no statistical difference between the L-DHA group and the H-DHA group. Moreover, the serum glucose, TP and TC levels were similar among the three groups.

**Table 1:** Effect of maternal DHA supplementation during lactation on serum biochemical indexes in weaning mice.

	Control	L-DHA	H-DHA	*p* value
Glucose (mmol/L)	4.882 ± 0.338	4.831 ± 0.360	4.932 ± 0.421	0.9821
TP (g/L)	38.110 ± 2.670	38.681 ± 2.232	37.026 ± 1.851	0.7888
TC (mmol/L)	1.970 ± 0.088	2.025 ± 0.098	2.071 ± 0.083	0.7301
TG (mmol/L)	1.151 ± 0.045^a^	1.488 ± 0.089^b^	1.564 ± 0.066^b^	0.0023

Data were expressed as the mean ± SEM; *n* = 8–10 mice per group. Statistical analysis was performed using one-way ANOVA test. Labeled means without a common letter differ at *p* value < 0.05. L-DHA: 150 mg/(kg body weight day) DHA; H-DHA: 450 mg/(kg body weight day) DHA. DHA, docosahexaenoic acid; TC, total cholesterol; TG, total triglyceride, TP, total protein.

### 3.4 Maternal DHA supplementation during lactation facilitates intestinal growth and changes intestinal fatty acid composition of offspring mice

**Figure 3:**
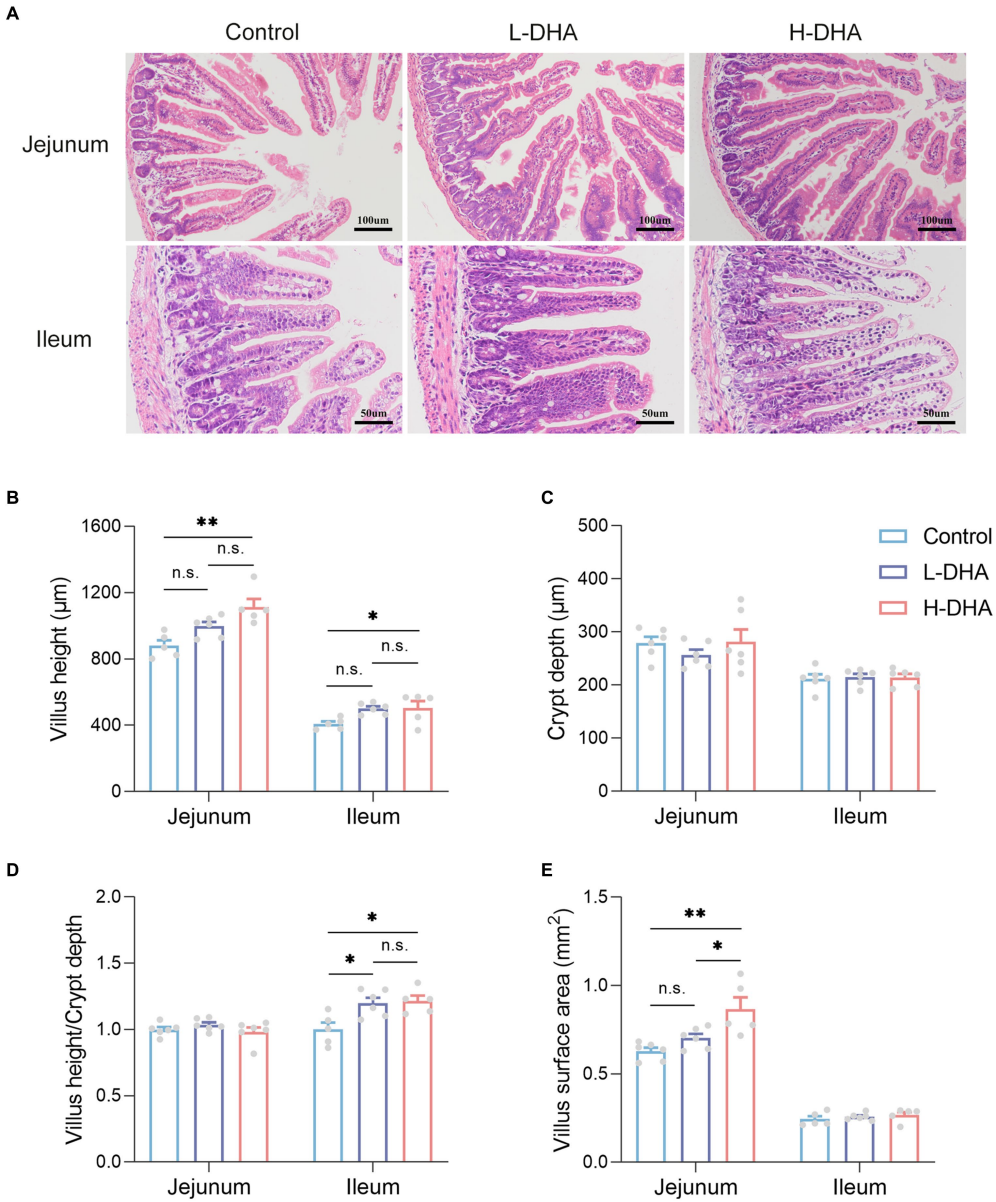
Effect of maternal DHA supplementation during lactation on small intestinal morphology in weaning mice. **(A)** Representative HE staining of jejunum (100x) and ileum (200x). **(B)** Quantitation of villus height of jejunum and ileum of mice from different groups. **(C)** Quantitation of crypt depth of jejunum and ileum of mice from different groups. **(D)** The ratio of villus height to crypt depth of jejunum and ileum of mice from different groups. **(E)** Quantitation of villus surface area of jejunum and ileum of mice from different groups. Data were expressed as the mean ± SEM; *n* = 5–6 per group. Significance was set at *p* < 0.05 (^*^p < 0.05, ^**^p < 0.01, ^***^p < 0.001). L-DHA: 150 mg/(kg body weight · day) DHA; H-DHA: 450 mg/(kg body weight · day) DHA. DHA, docosahexaenoic acid.

It is well known that small intestine is the main site for nutrient absorption. To characterize the effect of maternal DHA supplementation on the morphology of the developing small intestine, we compared jejunum and ileum morphology among groups. Representative H&E-stained jejunal and ileal images of the three groups were shown in Figure 3A. We observed a significant increase in jejunal and ileal villus height in the H-DHA group, a 0.27-fold increase in jejunal villus height (*p* < 0.01) and a 0.24-fold increase in ileal villus height (*p* < 0.05), as compared with the control group. Although jejunal and ileal villus height in the L-DHA group was also increased, it did not reach statistical significance compared to the control group (Figure 3B). The ratio of villus height to crypt depth in the ileum was increased by 20 and 21% in the L-DHA and H-DHA groups compared with the control group (*p* < 0.05 and *p* < 0.05, respectively) (Figure 3D). In addition, the jejunal villus surface in the H-DHA group resulted in a 0.38-fold increase (*p* < 0.01) when compared with the control group and resulted in a 0.23-fold increase (*p* < 0.05) when compared with the L-DHA group (Figure 3E). However, the crypt depth of jejunum and ileum, the ratio of villus height to crypt depth of jejunum and villus surface of ileum were similar among the three groups (Figures 3C–E).

We also investigated whether maternal DHA supplementation could change the fatty acid composition of the offspring jejunum. Our findings revealed that the relative levels of eicosapentaenoic acid, DHA, and n-3 PUFAs in the jejunal tissue were significantly elevated (11-fold, 2.8-fold, and 1.6-fold increase), while the relative level of stearic acid was significantly lower (40% decrease) in the H-DHA group compared to the control group (all *p* < 0.05). The detailed fatty acid compositions were provided in Table 2.

**Table 2:** Fatty acid compositions of jejunal tissue in weaning offspring.

Fatty acid	Control	L-DHA	H-DHA	*p* value
*Percentage of total fatty acid*				
C14:0	1.71 ± 0.44	1.01 ± 0.25	1.23 ± 0.33	0.382
C16:0	29.39 ± 1.99	26.39 ± 1.65	25.39 ± 2.76	0.431
C16:1n-9	7.71 ± 1.85	8.62 ± 2.19	9.49 ± 1.79	0.816
C18:0	21.33 ± 3.02^a^	19.27 ± 1.58^a^	12.56 ± 1.47^b^	0.034
C18:1n-9	18.77 ± 1.77	17.73 ± 3.33	13.24 ± 1.24	0.234
C18:2n-6	0.67 ± 0.21	0.61 ± 0.23	0.83 ± 0.22	0.777
C18:3n-3 (ALA)	1.21 ± 0.26^a^	3.11 ± 0.48^b^	2.04 ± 0.61	0.045
C20:0	2.62 ± 0.35	1.95 ± 0.41	2.20 ± 0.51	0.551
C20:4n-6 (AA)	3.41 ± 0.66	3.16 ± 0.71	4.57 ± 1.55	0.613
C20:5n-3 (EPA)	0.20 ± 0.09^a^	0.46 ± 0.16^a^	2.21 ± 0.59^b^	0.004
C22:4n-6	0.38 ± 0.13	0.52 ± 0.24	0.50 ± 0.18	0.862
C22:5n-3 (DPA)	0.57 ± 0.15	0.71 ± 0.29	0.59 ± 0.09	0.883
C22:6n-3 (DHA)	0.67 ± 0.19^a^	0.54 ± 0.16^a^	2.58 ± 0.71^b^	0.011
Total SFA	55.06 ± 5.02	48.61 ± 2.32	41.39 ± 2.68	0.056
Total MUFA	26.48 ± 1.74	26.35 ± 1.91	22.73 ± 2.28	0.353
n-3 PUFA	2.67 ± 0.39^a^	4.81 ± 0.74^a^	7.42 ± 0.92^b^	0.002
n-6 PUFA	4.46 ± 0.77	4.29 ± 0.65	5.91 ± 1.47	0.498

Data were expressed as the mean ± SEM; n = 5 mice per group. Statistical analysis was performed using one-way ANOVA test. Labeled means without a common letter differ at P value < 0.05. L-DHA: 150 mg/(kg body weight day) DHA; H-DHA: 450 mg/(kg body weight day) DHA. AA, arachidonic acid; ALA, α-linolenic acid; DHA, docosahexaenoic acid; DPA, docosapentaenoic acid; EPA, eicosapentaenoic acid; MUFA, monounsaturated fatty acids; n, omega; PUFA, polyunsaturated fatty acids; SFA, saturated fatty acids.

### 3.5 Effects of maternal DHA supplementation during lactation on fecal microbiota of offspring mice

**Figure 4:**
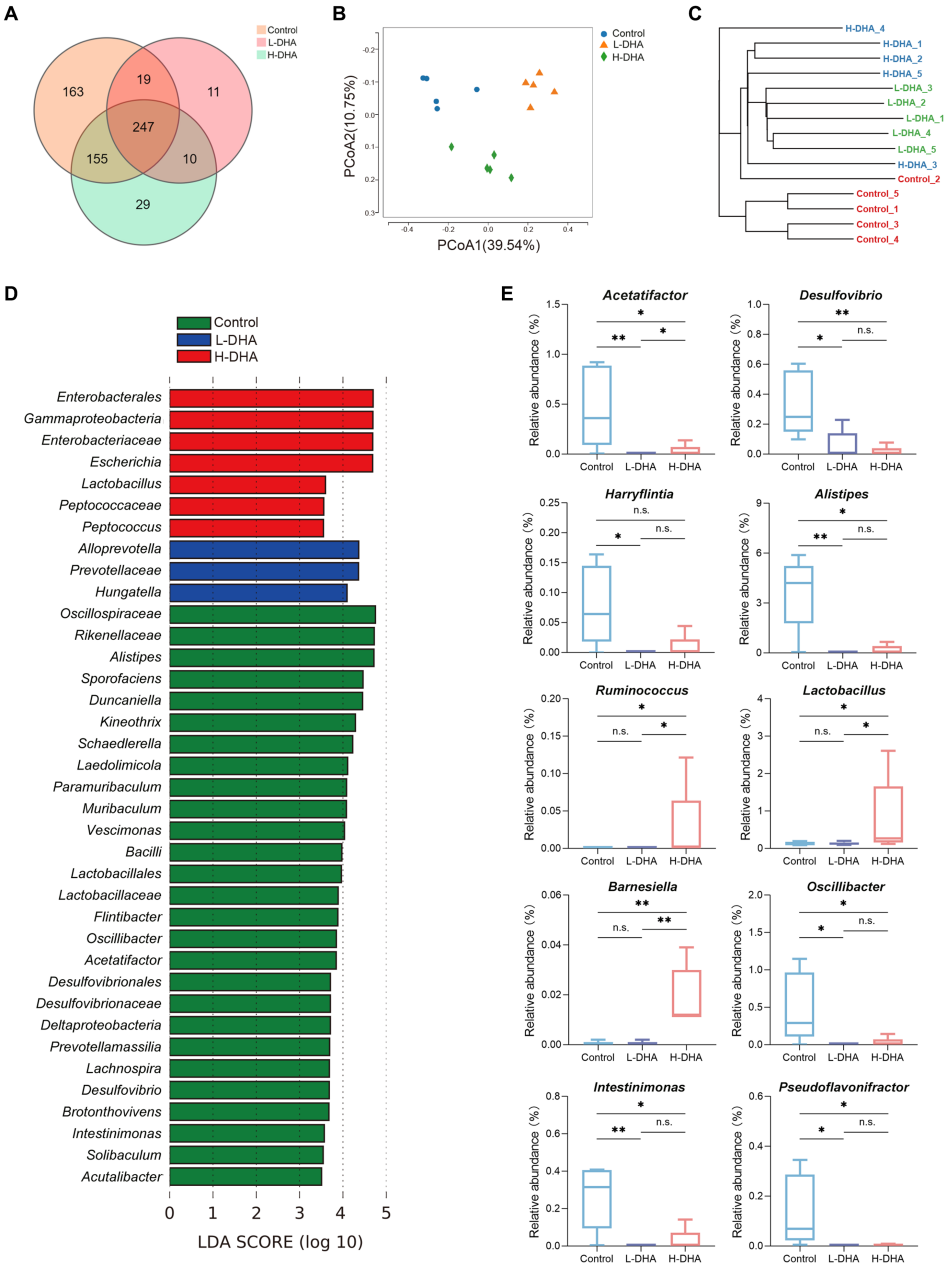
Effect of maternal DHA supplementation during lactation on the gut bacterial structure of weaning mice. **(A)** Venn diagram of the OTUs in different groups. **(B)** PCoA plot based on unweighted Unifrac distance in different groups. **(C)** UPGMA cluster tree based on unweighted Unifrac distance in different groups. **(D)** LEfSe analysis (LDA score ≥ 3.5) of the different gut microbiota in the genus level among three groups. **(E)** Significantly different genera in different groups. *n* = 5 per group. Significance was set at *p* < 0.05 (^*^p < 0.05, ^**^p < 0.01). L-DHA: 150 mg/(kg body weight day) DHA; H-DHA: 450 mg/(kg body weight · day) DHA. DHA, docosahexaenoic acid; OUT, operational taxonomic units; PCoA, principal coordinates analysis; UPGMA, unweighted pair group method with arithmetic mean; LEFSe, Linear discriminant analysis effect size; LDA, Linear discriminant analysis.

16 s rRNA sequencing was conducted in five fecal samples from each group to explore the effect of maternal DHA supplementation during lactation on the gut bacterial structure of pups. The overlapping and unique OTUs among groups were displayed by Venn diagram (Figure 4A). *β*-diversity analyses, PCoA and UPGMA based on unweighted Unifrac distance, indicated that the overall microbial compositions in the control group and the DHA-treated groups separated from each other (Figures 4B,C). As shown in Supplementary Figure S1A, compared with the control group, the L-DHA group exhibited a significantly lower Chao index (*p* < 0.01) and the H-DHA group also showed a decreased Chao index. However, no differences were found in Simpson index and Shannon index among the three groups. Next, we calculated the relative abundance of bacterial taxa at the phylum and genera level in each pup among the three groups (Supplementary Figures S1B,C). At the phylum level, *Bacillota* and *Bacteroidetes* were the dominant bacteria in the gut microbiota of weaning mice, and the L-DHA and H-DHA groups compared with the control group insignificantly decreased the relative abundance of *Bacillota*, while insignificantly increased that of *Bacteroidetes*. The ratios of *Bacillota*/*Bacteroidetes* were reduced in the L-DHA and H-DHA groups but did not reach statistical significance when compared with the control group (Supplementary Figure S1D). LEfSe analysis demonstrated significantly different microbiota among the groups at the genus levels (Figure 4D). In addition, maternal DHA supplementation during lactation decreased the relative abundance of some harmful bacteria and increased the relative abundance of several beneficial bacteria at the genus level. The relative abundance of *Acetatifactor* (*p* < 0.01), *Desulfovibrio* (*p* < 0.05), *Harryflintia* (*p* < 0.05), *Alistipes* (*p* < 0.01), *Oscillibacter* (*p* < 0.05), *Intestinimonas* (*p* < 0.01) and *Pseudoflavonifractor* (*p* < 0.05) were distinctly lower in the L-DHA group than those in the control group. Compared to the control group, the relative abundance of *Acetatifactor* (*p* < 0.05), *Desulfovibrio* (*p* < 0.01), *Alistipes* (*p* < 0.05), *Oscillibacter* (*p* < 0.05), *Intestinimonas* (*p* < 0.05) and *Pseudoflavonifractor* (*p* < 0.05) were significantly decreased, while the relative abundance of *Ruminococcus* (*p* < 0.05), *Lactobacillus* (*p* < 0.05) and *Barnesiella* (*p* < 0.01) were markedly increased in the H-DHA group (Figure 4E).

**Figure 5:**
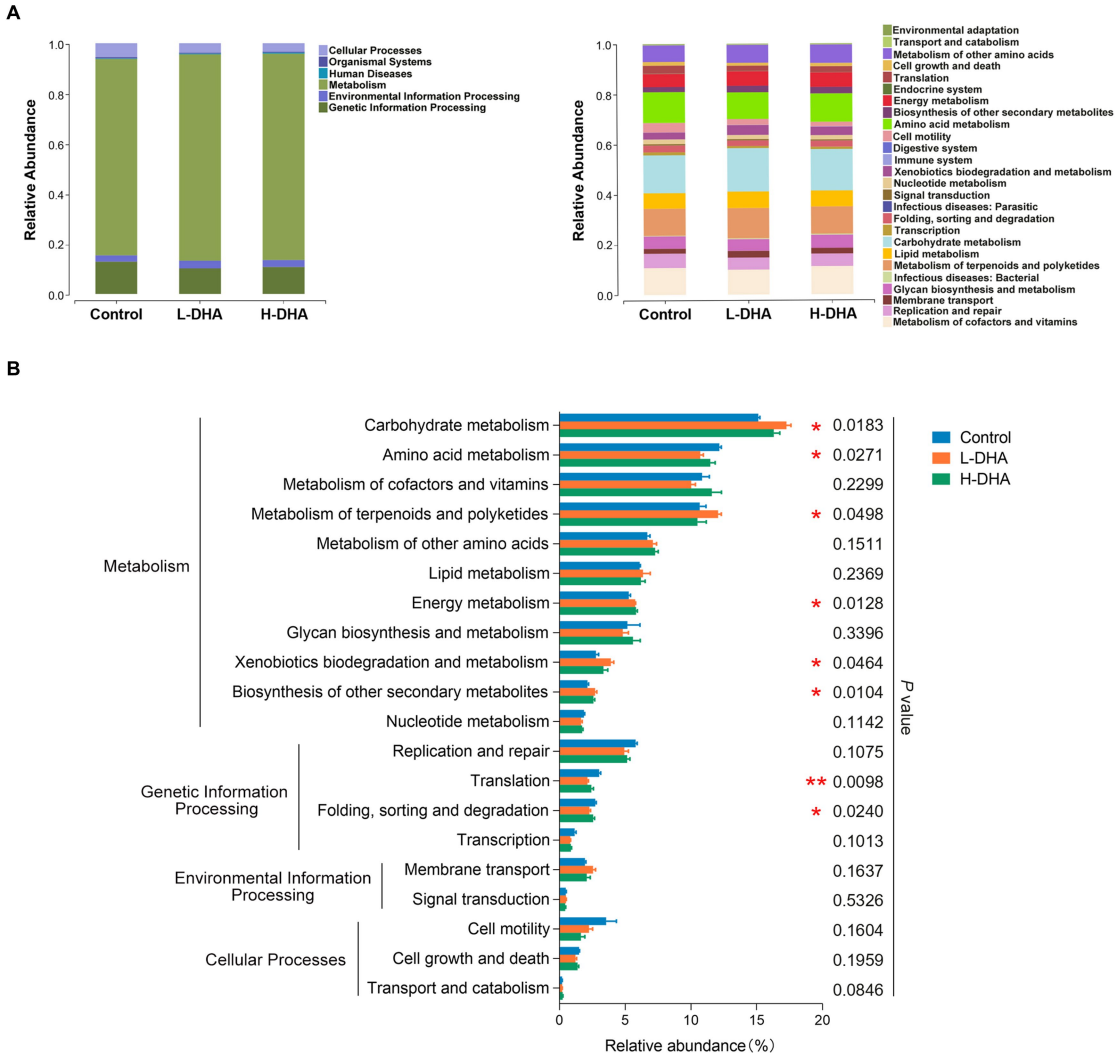
PICRUST functional prediction based on KEGG database in weaning mice. **(A)** Relative functional abundance at levels 1 and 2 among the control group, L-DHA group and H-DHA group. **(B)** Comparisons of KEGG pathways among the control group, L-DHA group and H-DHA group. *n* = 5 per group. Significance was set at *p* < 0.05 (^*^p < 0.05, ^**^p < 0.01). L-DHA: 150 mg/(kg body weight · day) DHA; H-DHA: 450 mg/(kg body weight day) DHA. DHA, docosahexaenoic acid; KEGG, Kyoto Encyclopedia of Genes and Genomes.

We further conducted functional prediction analysis based on KEGG database to understand the potential difference in fecal microbiota among groups. Functional compositions of the three groups at level 1 and 2 were shown in Figure 5A. Compared with the control group, the DHA-treated groups exhibited up-regulated trend in the abundances of pathways involved in carbohydrate metabolism (*p* < 0.05), metabolism of terpenoids and polyketides (*p* < 0.05), energy metabolism (*p* < 0.05), xenobiotics biodegradation and metabolism (*p* < 0.05) and biosynthesis of other secondary metabolites (*p* < 0.05), and down-regulated trend in the abundances of pathways related to amino acid metabolism (*p* < 0.05), translation (*p* < 0.01) and folding, sorting and degradation (*p* < 0.05). Intriguingly, most of these altered pathways were mainly prominent in the L-DHA group (Figure 5B).

### 3.6 Maternal DHA supplementation during lactation alters jejunum proteome of offspring mice

**Figure 6:**
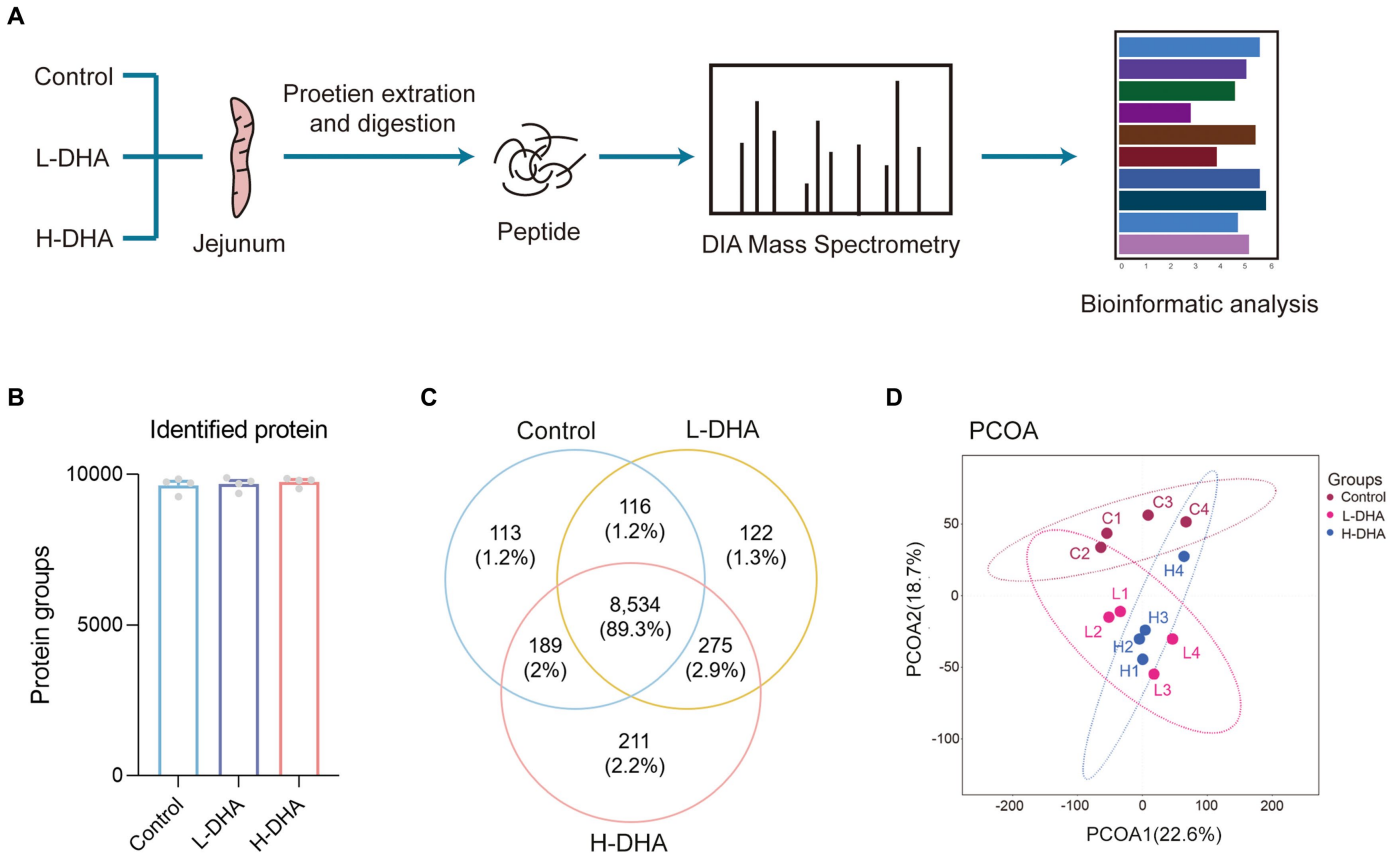
The workflow and profiling of weaning mice jejunum proteomes using LC–MS/MS. **(A)** Schematic diagram of LC–MS/MS experimental procedure in this study. **(B)** The total identified protein number in each group. **(C)** Venn diagram showing the total numbers of identified proteins among the three groups. **(D)** PCoA of all groups. Data were expressed as the mean ± SEM; *n* = 4 per group. L-DHA: 150 mg/(kg body weight day) DHA; H-DHA: 450 mg/(kg body weight · day) DHA. DHA, docosahexaenoic acid; PCoA, principal coordinates analysis.

In order to understand the underlying biological effects of maternal DHA supplementation during lactation on the intestinal development of pups, we analyzed the proteomics of jejunum tissues from three groups with the DIA-MS method (Figure 6A). There were 9,641, 9,681, and 9,754 identified proteins and 113, 122, and 211 unique proteins in the control, L-DHA, and H-DHA groups, respectively, while 8,534 proteins overlapped among the three groups (Figures 6B,C). PCoA analysis showed that the control, L-DHA, and H-DHA groups were separated from each other (Figure 6D). Kernel density plot and correlation analysis between samples were presented in Supplementary Figures S2A,B.

**Figure 7:**
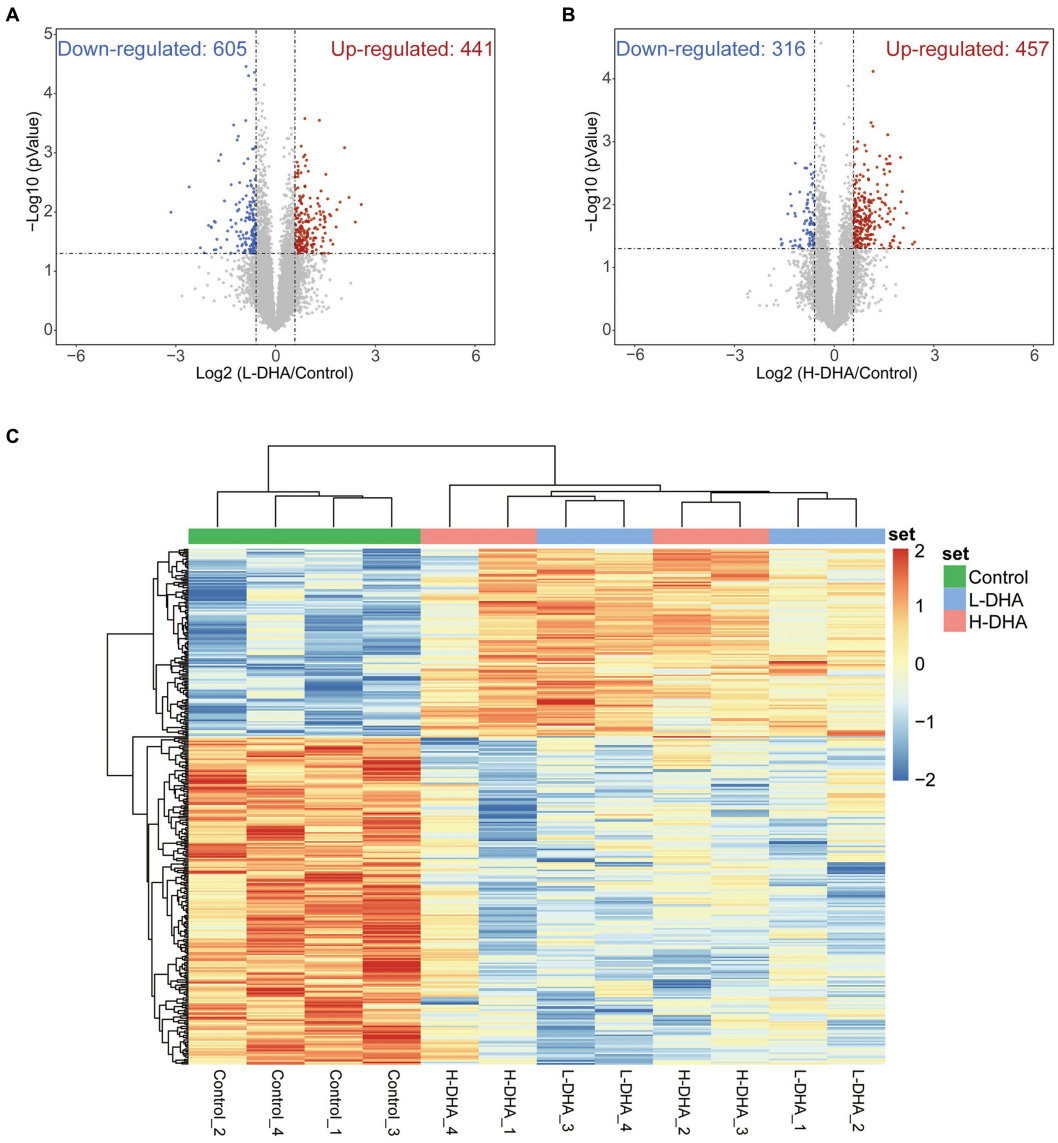
Identification of differentially expressed proteins in weaning mice. **(A)** Volcano plot of up-regulation or down-regulation of differentiated proteins for the L-DHA group compared with the control group. **(B)** Volcano plot of up-regulation or down-regulation of differentiated proteins for the H-DHA group compared with the control group. Red points represent up-regulated proteins in the L-DHA or H-DHA group, blue points represent down-regulated proteins in the L-DHA or H-DHA group (fold change >1.5, *p* value <0.05) and gray points represent unchanged proteins. **(C)** The heat map of hierarchical clustering analysis on the differentially expressed proteins in the three groups. *n* = 4 per group. L-DHA: 150 mg/(kg body weight day) DHA; H-DHA: 450 mg/(kg body weight day) DHA. DHA, docosahexaenoic acid.

Subsequently, the jejunal proteome profiles of the L-DHA and H-DHA groups were compared with those of the control group, individually. A total of 1,046 differentially expressed proteins (FC > 1.5, *p* < 0.05) were determined between the control group and the L-DHA group, among which 441 proteins were up-regulated and 605 proteins were down-regulated in the L-DHA group (Figure 7A). There were 873 differentially expressed proteins (FC > 1.5, *p* < 0.05) in total between the control group and the H-DHA group, and 457 proteins up-regulated and 316 proteins down-regulated in the H-DHA group (Figure 7B). Next, hierarchical clustering heat map showed the expression tendency for these differentially expressed proteins. The patterns of differentially expressed proteins were found to be similar in the L-DHA and H-DHA groups, while both were significantly different from the pattern in the control group (Figure 7C).

**Figure 8:**
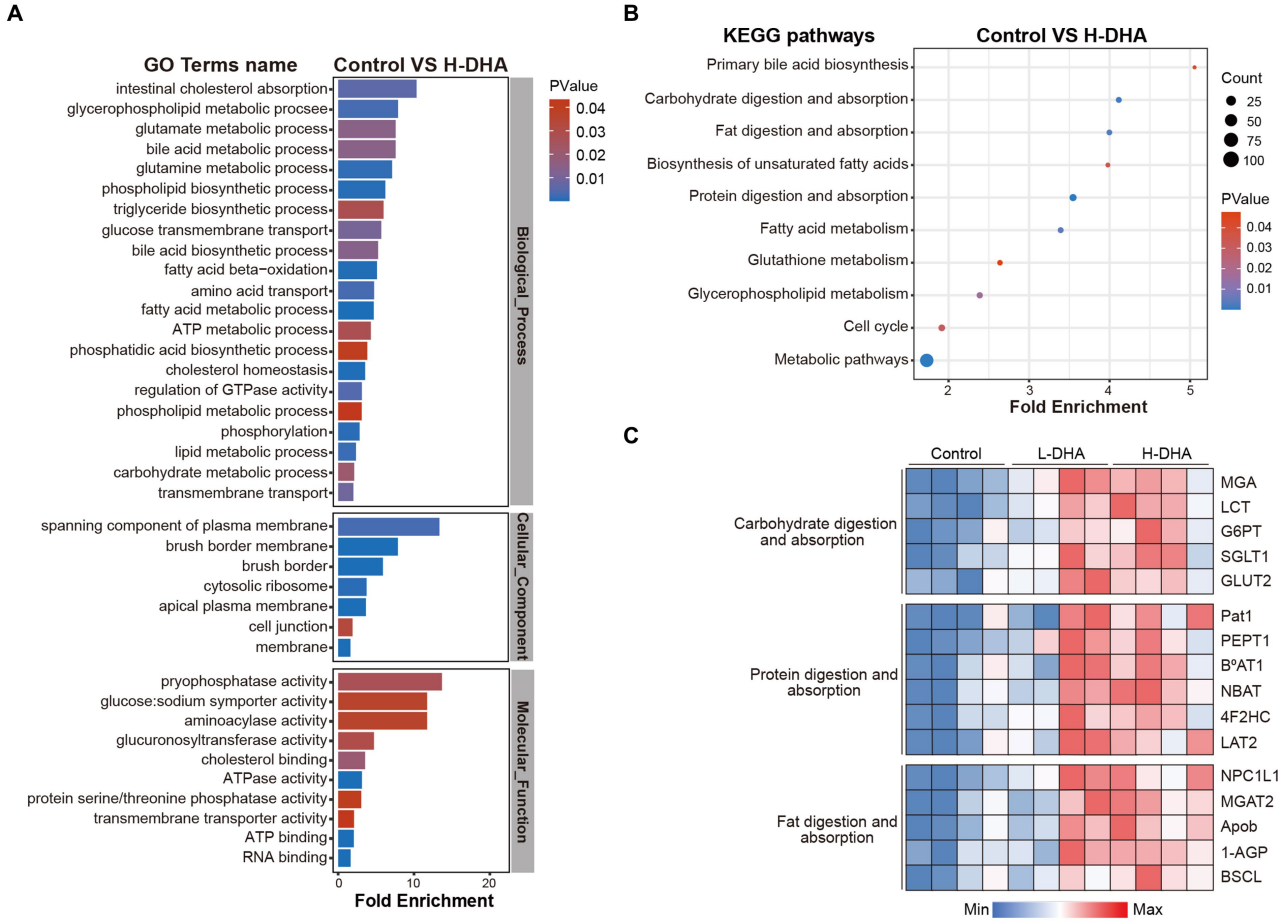
Effects of maternal DHA supplementation during lactation on the jejunum proteomic level in weaning mice. **(A)** GO analysis and **(B)** KEGG pathways analysis in control and H-DHA groups. **(C)** Heat map analysis based on the intensity of representative proteins in classifications related to nutritional absorption and metabolism for the control, L-DHA, and H-DHA groups. *n* = 4 per group. L-DHA: 150 mg/(kg body weight day) DHA; H-DHA: 450 mg/(kg body weight day) DHA. DHA, docosahexaenoic acid; GO, Gene ontology; KEGG, Kyoto Encyclopedia of Genes and Genomes.

To further identify the effects of maternal DHA supplementation, GO enrichment analysis, including biological process (BP) analysis, molecular function (MF) analysis and cell component (CC) analysis, and KEGG pathway enrichment analysis were performed using differentially expressed proteins when the control group was compared with the L-DHA group or the H-DHA group, respectively. As shown in Figure 8A, between the control and H-DHA groups, BP analysis revealed that the metabolic and transport-related processes were significantly affected; terms mainly related to membrane, especially brush border membrane, were enriched in CC analysis; MF analysis showed that glucose: sodium symporter activity was significantly enriched. Meanwhile, KEGG pathways analysis showed that carbohydrate digestion and absorption, fat digestion and absorption and protein digestion and absorption were significantly enriched (Figure 8B). Between the control and L-DHA groups, enrichment in CC analysis was similar to the results between the control and H-DHA groups, and protein digestion and absorption was also enriched in KEGG pathways analysis, but carbohydrate digestion and absorption as well as fat digestion and absorption were not affected (Supplementary Figures S2C,D). Heat map analysis on the representative proteins associated with nutrient absorption and metabolism in transport-related classifications among the three groups suggested that the abundance of these proteins was increased in DHA-treated groups (Figure 8C). These results indicated that maternal DHA supplementation during lactation may improve the capacity of jejunum absorption and transport in weaning mice.

### 3.7 Maternal DHA supplementation during lactation enhances the expressions of jejunal glucose transport-associated proteins of offspring mice

**Figure 9:**
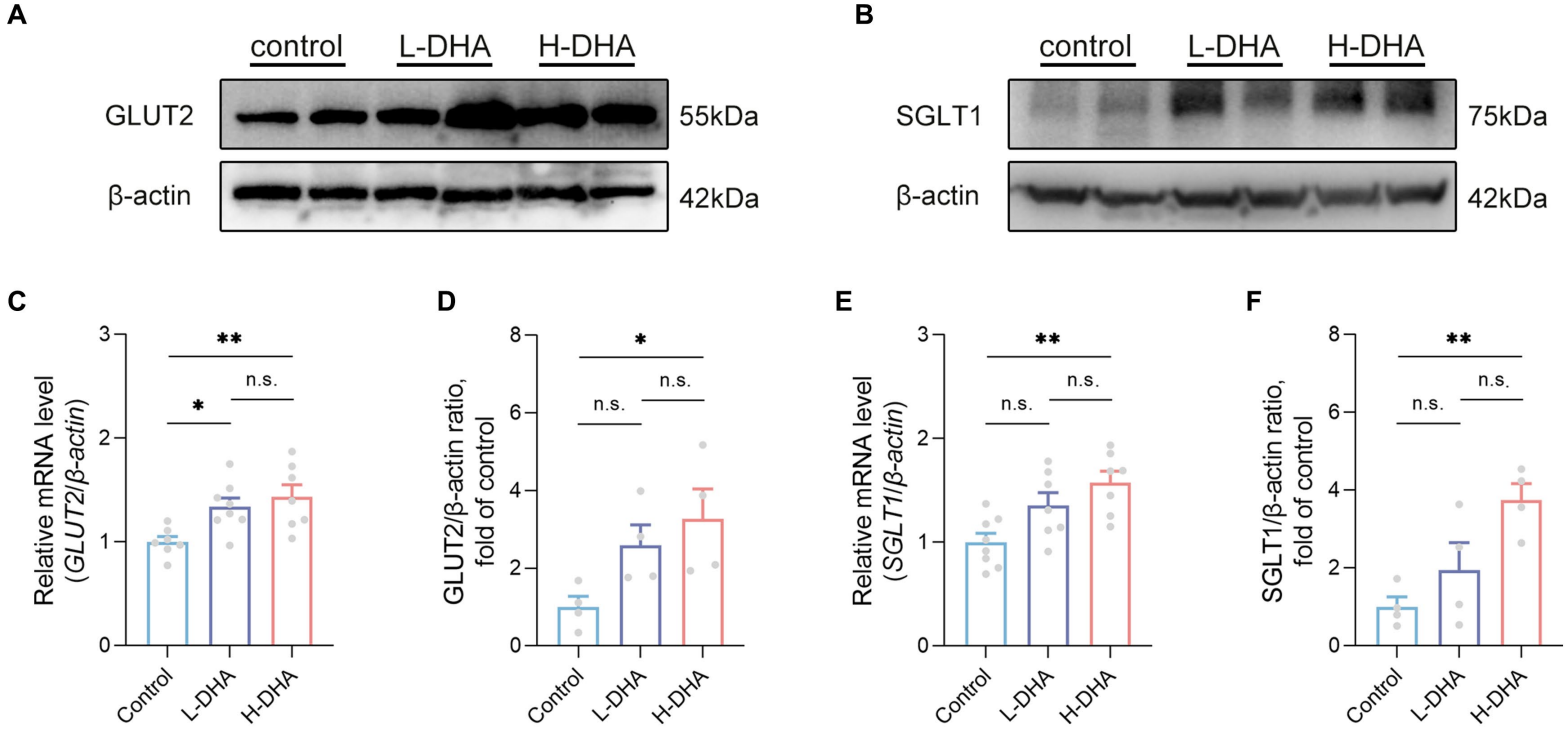
Effect of maternal DHA supplementation during lactation on the jejunal glucose transport-associated gene expression in weaning mice. Representative Western blots of **(A)** GLUT2 and **(B)** SGLT1, and **(D,F)** their relative band intensities in jejunal tissue of mice from different groups. n = 4 per group. Relative mRNA levels of **(C)** *GLUT2* and **(E)** *SGLT1* in jejunal tissue of mice from different groups. *n* = 7–8 per group. Data were expressed as the mean ± SEM. Significance was set at *p* < 0.05 (^*^p < 0.05, ^**^p < 0.01). L-DHA: 150 mg/(kg body weight day) DHA; H-DHA: 450 mg/(kg body weight · day) DHA. DHA, docosahexaenoic acid; GLUT2, glucose transporter 2; SGLT1, sodium glucose co-transporter 1.

We further detected the expressions of genes related to glucose absorption and transport in the jejunum. Western blot analysis indicated that the expression of GLUT2 protein in the H-DHA group was significantly increased by 227% compared with the control group (Figures 9A,D; *p* < 0.05). Additionally, the *GLUT2* mRNA level in the L-DHA group and H-DHA group was 34 and 43% greater than that in the control group (Figure 9C; *p* < 0.05 and *p* < 0.01, respectively). The protein and mRNA expressions of SGLT1 in the jejunum were notably 275 and 57% higher in the H-DHA group than that in the control group (Figures 9B,E,F; both *p* < 0.01). Although not reaching statistical significance, the L-DHA group tended to elevate the mRNA expression of *SGLT1* (36% increase) and the protein expressions of GLUT2 (159% increase) and SGLT1 (94% increase), as compared with the control group (Figures 9D–F; *p* = 0.0661, *p* = 0.1685 and *p* = 0.0711, respectively). Meanwhile, we also determined the mRNA expressions of genes involved in lipid and protein transport in the jejunum, and the results showed that maternal DHA supplementation significantly modulated some of them. In the L-DHA and H-DHA groups, compared with the control group, the mRNA expression of *NPC1L1* was increased by 97 and 113% (Supplementary Figure S3A; *p* < 0.05 and *p* < 0.01, respectively), the mRNA expression of *CD36* was 87 and 101% greater (Supplementary Figure S3A; *p* < 0.01 and *p* < 0.001, respectively), and the mRNA level of *ASCT2* was up-regulated by 82 and 72% (Supplementary Figure S3B; both *p* < 0.05). Besides, compared with both the control group and L-DHA group, the H-DHA group exhibited a remarkable 234% and a 64% increase in the mRNA expression of *APOB* (Supplementary Figure S3A; *p* < 0.001 and *p* < 0.05, respectively). Intriguingly, the mRNA level of *APOA1* was 52 and 48% lower in the L-DHA group and the H-DHA group than in the control group (Supplementary Figure S3A; both *p* < 0.01).

### 3.8 Maternal DHA supplementation during lactation increases intestinal glucose absorption and tissue glycogen storage of offspring mice

**Figure 10:**
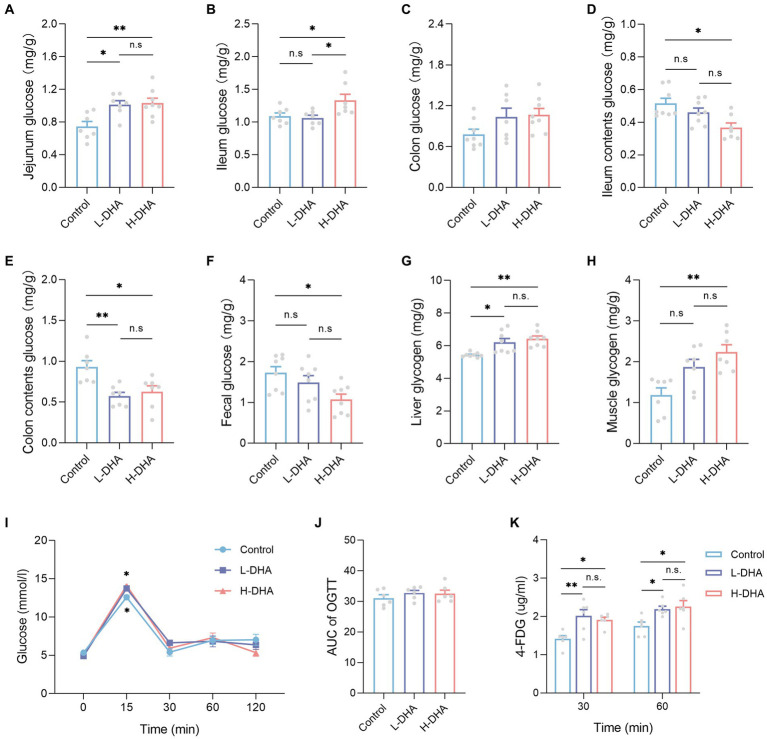
Effect of maternal DHA supplementation during lactation on intestinal glucose absorption and tissue glycogen storage in weaning mice. **(A-H)** Mice untreated with glucose and 4-FDG before sacrifice. *n* = 7–8 per group. The levels of **(A)** jejunum glucose, **(B)** ileum glucose, **(C)** colon glucose, **(D)** ileum contents glucose, **(E)** colon contents glucose, **(F)** feces glucose, **(G)** liver glycogen, and **(H)** muscle glycogen in weaning mice. **(I-K)** 2 g/kg glucose and 10 mg/kg 4-FDG were orally co-administered followed by blood glucose and 4-FDG quantitation at various time points. *n* = 6 per group. **(I)** OGTT and **(J)** AUC of weaning mice subjected to glucose/4-FDG oral gavage. **(K)** Serum 4-FDG levels of weaning mice subjected to glucose/4-FDG oral gavage. Data were expressed as the mean ± SEM. Significance was set at *p* < 0.05 (^*^p < 0.05, ^**^p < 0.01). L-DHA: 150 mg/(kg body weight day) DHA; H-DHA: 450 mg/(kg body weight day) DHA. DHA, docosahexaenoic acid; OGTT, oral glucose tolerance test; AUC, area under the curve; 4-FDG, 4-deoxy-4-fluoro-D-glucose.

We observed a significant increase in jejunum glucose concentrations in the L-DHA and H-DHA groups, a 0.36-fold increase and a 0.38-fold increase individually, as compared with the control group (Figure 10A; *p* < 0.05 and *p* < 0.01, respectively). The ileum glucose concentration in the H-DHA group was 22% greater than in the control group, and 25% higher than in the L-DHA group (Figure 10B; both *p* < 0.05). Notwithstanding, no significant difference in colon glucose concentrations was found among groups (Figure 10C). The glucose concentrations of ileum contents (29% decrease), colon contents (33% decrease) and feces (42% decrease) in the H-DHA group were dramatically lower than in the control group (*p* < 0.05, *p* < 0.05 and *p* < 0.01, respectively); the colon contents glucose was also strongly reduced by 38% in the L-DHA group when compared with the control group (*p* < 0.01), whereas there were no significant differences in glucose concentrations of ileal contents and feces (Figures 10D–F). Furthermore, the glycogen levels of the liver and muscle were 0.17-fold and 0.88-fold higher in the H-DHA group than those in the control group (Figures 10G,H; both *p* < 0.01). In the L-DHA group, the liver glycogen concentration was increased by 13% compared to the control group (Figure 10G; *p* < 0.05), and the muscle glycogen level also exhibited a 57% increase, although not reaching statistical difference (Figure 10H).

To further demonstrate the reliability of our outcomes, the glucose and 4-FDG absorption test was conducted *in vivo*, using a co-load of 2 g/kg glucose and 10 mg/kg 4-FDG. According to the oral glucose tolerance test (OGTT), the blood glucose levels were higher at 15 min in the L-DHA group and the H-DHA group than in the control group (Figure 10I; both *p* < 0.05), but there was no significant difference in the AUC of OGTT among groups (Figure 10J). Compared with the control group, the L-DHA group increased the concentrations of 4-FDG at 30 and 60 min (Figure 10K; *p* < 0.01 and *p* < 0.05, respectively). Similarly, the H-DHA group exhibited higher serum 4-FDG contents than those in the control group (Figure 10K; both *p* < 0.05).

### 3.9 Maternal DHA supplementation during lactation up-regulates the mTOR signaling pathway of offspring mice

**Figure 11:**
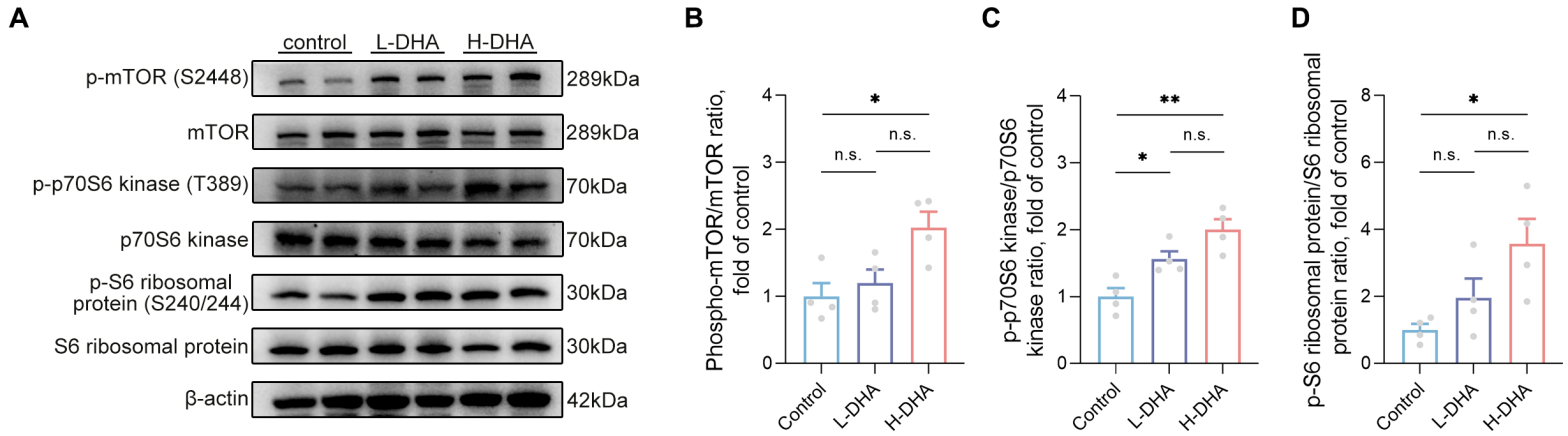
Effect of maternal DHA supplementation during lactation on the mTOR signaling pathway in weaning mice. **(A)** Representative Western blots of p-mTOR (S2448), total mTOR, p-p70S6 kinase (T389), total p70S6 kinase, p-S6 ribosomal protein (S240/244) and total S6 ribosomal protein, and **(B, C, D)** the relative band intensities of phosphorylation levels normalized to their respective total proteins in jejunal tissue of mice from different groups. Data were expressed as the mean ± SEM; *n* = 4 per group. Significance was set at *p* < 0.05 (^*^p < 0.05, ^**^p < 0.01). L-DHA: 150 mg/(kg body weight day) DHA; H-DHA: 450 mg/(kg body weight day) DHA. DHA, docosahexaenoic acid; mTOR, mammalian target of rapamycin.

Phosphorylation levels of mTOR and downstream p70S6 kinase and S6 ribosomal protein were determined to further explore the possible molecular mechanism underlying the effect of maternal DHA supplementation during lactation to promote intestinal glucose uptake in pups. The results revealed that the phosphorylation of mTOR (S2448), p70S6 kinase (T389) and S6 ribosomal protein (S240/244) were notably up-regulated in the H-DHA group, 103% increase, 100% increase and 257% increase respectively, compared with the control group (Figures 11A–D; *p* < 0.05, *p* < 0.01 and *p* < 0.05, respectively). A marked 56% higher level of phosphorylation of p70S6 kinase (T389) was observed in the L-DHA group than in the control group (Figure 11C; *p* < 0.05), whereas no differences were found in the phosphorylation levels of mTOR (S2448) and S6 ribosomal protein (S240/244) between the control and L-DHA groups.

## 4 Discussion

Early life nutrition plays a crucial role in shaping the development of bodily systems and programming the gut microbiota, ultimately influencing lifelong health (25, 26). The transition from suckling to weaning is a pivotal period prone to intestinal dysfunction and dysbiosis, often resulting in growth retardation. Therefore, mitigating the adverse effects of weaning stress on offspring has become a significant public health concern. Nowadays, perinatal maternal nutrition is gaining increased attention due to its close association with the early nutrition of offspring. DHA, produced only in marginal amounts by the body, is recognized as a vital nutrient crucial for visual and brain development. Daily supplementation with DHA is a common clinical practice for perinatal women (7, 8). Despite numerous studies focusing on maternal DHA supplementation during pregnancy and lactation, very few researches have investigated its impact on gut development, including the underlying biochemical and molecular mechanisms. In this study, we found that maternal DHA feeding improved exercise performance, modulated gut microbiota, increased intestinal glucose absorption capacity and enhanced tissue glycogen storage in pups.

Considering the mice used in the present study were normal and non-defective, the effect of maternal DHA supplementation on the body and organ weights of the pups was likely to be negligible. Numerous studies have reported several beneficial effects of fish oil and DHA, such as increased muscle mass and function in healthy older adults following the oral administration of fish oil (27), and improved physical exercise performance in adult mice fed ethanol extract from the red seaweed *Gloiopeltis furcata*, in which DHA is the prominent active ingredient (28). It is essential to underscore that our investigation specifically addresses the influence of maternal DHA supplementation during lactation, distinguishing it from prior research that focused on DHA intake during pregnancy. The experimental design used in this study is based on Richard’s study on DHA supplementation during lactation, considering 1–2 days before parturition as the adaptation period for lactational exposure (29, 30). The parturition period for mice is GD19-GD21, and GD19 is considered full term for fetal mice. By GD18, the major organs of fetal mice are almost completely developed, and the treatment beginning at GD18 is expected to have limited impact on fetal mice at late pregnancy stage.

Notably, our findings reveal an enhancement in exercise performance among offspring with maternal DHA supplementation during lactation, aligning with distinct results from another study that demonstrated maternal DHA intake during pregnancy mitigating grip strength impairment induced by MeHg in mouse pups (31). This emphasizes the critical consideration of the specific timing of DHA supplementation. Physical fatigue primarily stems from energy expenditure and a deficiency of energy during exercise. Carbohydrates serve as the principal energy source when skeletal muscles engage in activity, primarily stored in the liver and muscles as glycogen, with a minor amount circulating in the bloodstream as glucose (32). During exercise, muscle glucose utilization is expedited and liver glycogen may break down to glucose, transported through the bloodstream to working muscles to support physical activity (33). Thereby, as the predominant source of glycolysis for energy production, maintaining optimal tissue glycogen concentrations in both muscle and liver is recognized as crucial for increasing endurance capacity and delaying the onset of fatigue (34). Our study indicated that maternal DHA supplementation during lactation could increase energy storage in mouse pups, providing a potential explanation for their improved exercise performance.

Although carbohydrate metabolism takes precedence, catabolized fat also could supply partial energy in the phase of exercise (34). TG would be hydrolyzed to free fatty acids and then provided for working muscles as fuel during exercise (35). In agreement with the research reported by Drouin et al., we found that the offspring from the DHA-treated groups also exhibited a higher serum TG, which may result from the content of DHA, mostly as the form of TG, was increased in milk after maternal DHA intake (30, 36). It is worth mentioning that, distinguished from adults, infants are in a period of negative energy balance and require greater lipid accumulation to support the high metabolic demands of physical development, especially for the development of the brain (37). Therefore, we deemed that the acceptable elevation of serum TG in our result did not adversely impact the pups.

During the early stages of life, gut maturation is essential to provide adequate nutritional support for growth development, and abnormal intestinal development commonly leads to feeding intolerance, impaired nutrient absorption and even necrotizing enterocolitis (38). Villus height, villus surface area and V/C ratios are widely used to reflect the healthy state of intestinal function and absorption in the small intestine and are correlated with the intestinal capacity to absorb and transport dietary nutrients (39). In the DHA-treated groups, we observed increased villus height, villus surface area as well as V/C ratios. Previous studies have reported that a diet supplemented with fish oil could mitigate LPS-induced intestinal morphology injury, evidenced by increased jejunal and ileal villus height and V/C ratios (40). Additionally, maternal fish oil supplementation has been shown to improve intestinal morphology in weaning pigs (41). DHA has also been demonstrated to ameliorate gut morphology injury following heatstroke (42). Unlike previous studies, our results demonstrated that maternal DHA supplementation during lactation may facilitate the intestinal absorption function of offspring mice under normal conditions, which provides a novel and viable intervention to promote infant gut development and health.

The gut microbiota is strongly associated with gut health and is involved in diverse functions of the host, including digestion, nutrition absorption, metabolism, pathogen protection, and immunity. There is growing evidence that supplementation with DHA is beneficial to gut flora (43, 44). The decreased richness index of gut microbiota in the L-DHA group is inconsistent with the study reported by Han et al., which demonstrated maternal DHA intake insignificantly affected the richness index of pups (14). Although not significant, our data showed a decreasing trend in the ratio of *Bacillota*/*Bacteroidetes* reported as a hallmark of obesity in the DHA-treated groups (45). Additionally, the relative abundance of specific bacteria changed significantly among the groups at the genus level. In the H-DHA group, the relative abundances of several genera, such as *Ruminococcus* associated with short-chain-fatty-acid-producing, *Lactobacillus* that improves gut integrity and counteracts endotoxemia and inflammation, and *Barnesiella* related to taurine-conjugated bile acids, were increased, which are consistent with previous studies (44, 46, 47). In contrast, we found the relative abundances of *Acetatifactor*, *Desulfovibrio*, *Harryflintia*, *Alistipes*, *Oscillibacter*, *Intestinimonas* and *Pseudoflavonifractor* identified as harmful inflammation-associated or obesity-associated bacteria were lower in the DHA-treated group (48–51). The decrease in the number of harmful bacteria was more than the increase in the number of beneficial bacteria, which may account for the reduction of the gut flora richness. The variations of *Desulfovibrio* and *Alistipes* agree with the studies reported by Ran et al. and Zhang et al. (52, 53), but the alterations of *Oscillibacter* and *Intestinimonas* are inconsistent with the results reported by Qian et al. and Zhuang et al. (54, 55). Furthermore, the KEGG functional prediction of fecal microbiota revealed that the metabolic pathways involved in carbohydrate metabolism and energy metabolism were up-regulated in the DHA-treated groups, suggesting an enhanced metabolism *in vivo*, which was conducive to growth and development in early life. In the present study, DHA maternal intervention showed an advantageous effect on programming the gut microbiota of pups.

The transition from breastfeeding to weaning is challenged by dietary changes that predispose to intestinal digestive and absorption disorders (12). There is mounting evidence that DHA could mainly promote neurogenesis and synaptogenesis in the brain (56, 57). However, our results revealed that maternal DHA supplementation during lactation also seemed to facilitate intestinal development in pups, which is different from the primary role of DHA found in previous studies. Therefore, we used emerging non-targeted proteomics to systematically investigate the potential biological effects of maternal DHA supplementation on the intestine of weaning mice. Previous study has reported that increased nutrient digestibility is associated with increased expression of enteric nutrient transporter protein genes (58). In the jejunal proteome results, KEGG and GO functional enrichment analyses that showed up-regulation of intestinal nutrients, including carbohydrate, lipid and protein, digestion and absorption indicated that maternal DHA supplementation during lactation affected jejunal membrane transport function, more pronounced in the H-DHA group, implying an improved nutrient transport and absorption capacity in the offspring, which was consistent with changes in intestinal morphology. It was further verified at the mRNA level that the expression of genes associated with glucose transport were all increased, whereas genes related to lipid and protein transport were only partially up- or even down-regulated. Meanwhile, the alterations in the functional annotation of the intestinal flora were primarily focused on carbohydrate metabolism, hence we speculated that DHA supplementation during lactation in dams more predominantly affected intestinal glucose absorption in the offspring. SGLT1 is responsible for the transport of glucose from the lumen to the epithelial cells, and subsequently promotes the glucose transporter protein, GLUT2, to mediate the transport of glucose through the basolateral membrane to the mesenchyme and thus into the blood circulation (59). Glucose uptake in the intestine is accomplished by the interplay of SGLT1 and GLUT2 in collaboration with each other. Moreover, we found the abundance of glucose transport proteins, both GLUT2 and SGLT1, in the jejunum of weaning offspring mice were enhanced in the DHA-supplemented groups, and there appeared to be a dose-dependent effect of this gain. Similar promotion of intestinal glucose absorption has been documented in previous studies where sows exposed to an n-3 LCPUFA enriched diet showed enhanced intestinal absorption in their weaning offspring (15, 60). However, our study more directly elucidates the role of maternal DHA intake during lactation and provides insights into the potentially effective dosage of supplementation.

Intestinal tissue is a pivotal organ in the regulation of glucose metabolism. We found that glucose levels were elevated in small intestinal tissues but decreased in intestinal contents and feces in the DHA-treated groups. Although commonly employed to assess insulin regulation of blood glucose, the OGTT is an important tool for assessing intestinal glucose absorption. Nevertheless, the OGTT is not a robust representation of intestinal glucose absorption capacity because it is related to the process of insulin regulation of blood glucose. The glucose analogue 4-FDG, a substrate for both GLUTs and SGLTs, is not metabolized *in vivo,* and could more exactly reflect intestinal glucose absorption (61). The data of our glucose uptake test *in vivo* further confirmed that maternal DHA supplementation could enhance intestinal glucose absorption in pups without disrupting glucose homeostasis. Glycogen, a branched polymer of glucose, acts as an energy reserve when nutritional sufficiency for consumption in times of need (62). Since gluconeogenesis and glucose utilization are expedited during exercise, high concentrations of muscle and liver glycogen prior to exercise are believed to be essential for exercise performance (33). Consistently, the increased muscle and liver glycogen contents in the DHA-supplemented groups indicated tissue energy storage in pups was enhanced, which in turn supported the outcomes of our behavioral tests.

**Figure 12:**
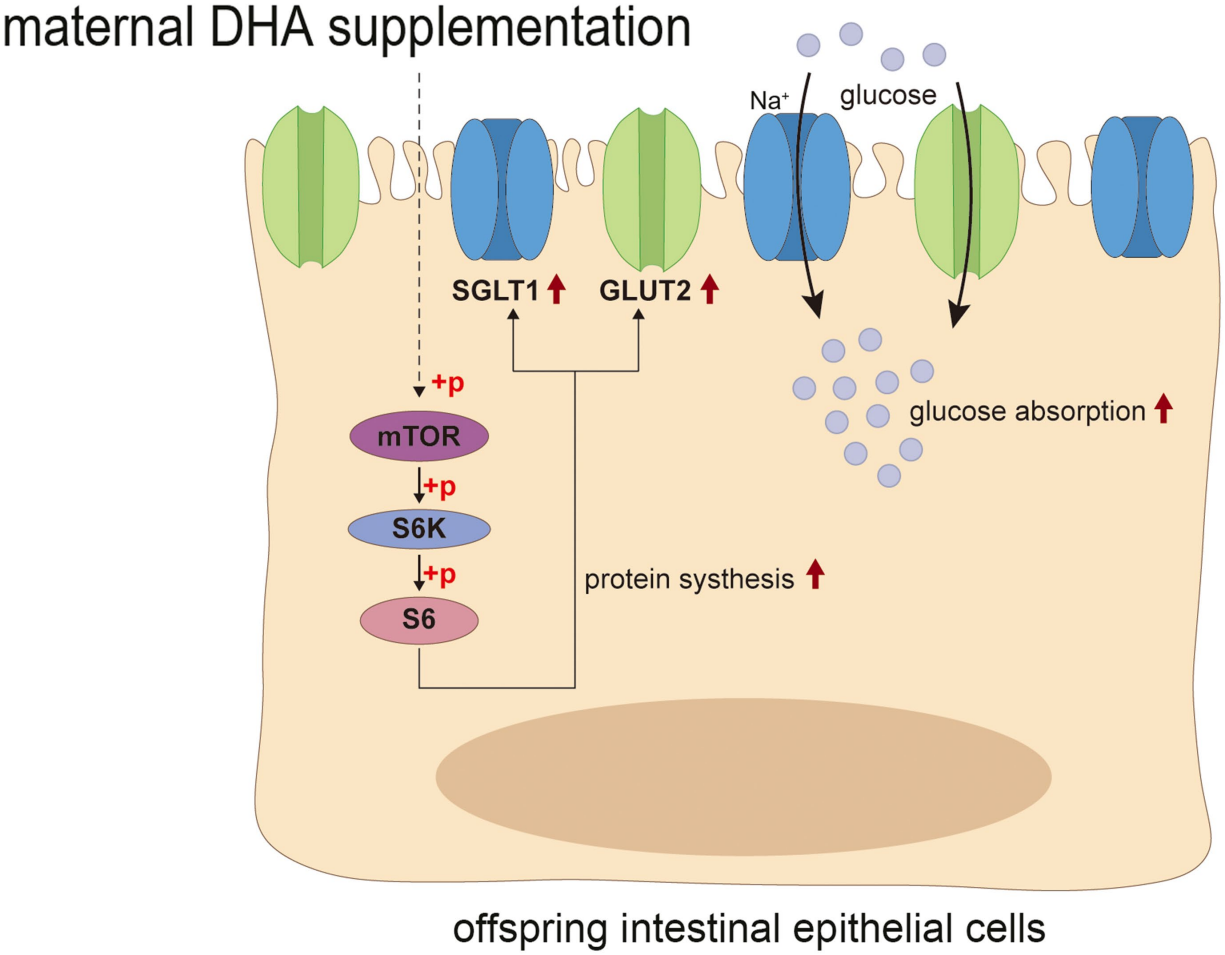
Possible mechanism of maternal DHA supplementation during lactation promotes intestinal glucose absorption in weaning mice. DHA, docosahexaenoic acid.

The mTOR signaling pathway is well known for its crucial function in regulating translation, protein synthesis and catabolism, nutrient sensing, energy utilization and growth factor signaling. It consists of two distinct multi-subunit complexes known as mTOR complex 1/2 (mTORC1/2) (63). Previous studies have shown that mTORC1-driven translational regulation is mainly dependent on catalyzing the phosphorylation of eukaryotic translation initiation factor 4E (eIF4E)-binding protein (4E-BP), p70S6 kinase, and its downstream players to enhance translation and protein synthesis, which is essential for mediating cellular proliferation and growth (64). Moreover, mTOR is believed to be closely linked to intestinal epithelium growth and proliferation. Systemic administration of rapamycin, a canonical mTOR inhibitor, has been shown to reduce intestinal surface area and disrupt intestinal regeneration (65). Activation of mTOR plays a critical role in promoting intestinal adaptation and regulating intestinal epithelial homeostasis and regeneration (65, 66). Notably, some studies suggested that the expression of GLUT2 could be regulated by the Akt/mTOR signaling pathway (67, 68). In the present study, we found that the improved intestinal tissue morphology, the enhanced intestinal glucose absorption, and the increased expression of glucose transport-associated proteins in offspring with maternal DHA supplementation may be attributed to the activation of the mTOR signaling pathway. This finding suggested that maternal DHA supplementation during lactation might modulate the mTOR signaling pathway in the intestines of breastfed offspring, thereby promoting intestinal glucose absorption (Figure 12). However, the specific mechanisms require further investigation.

## 5 Conclusion

In summary, the results presented in this work demonstrated that maternal DHA supplementation during lactation enhances the intestinal glucose absorption. This effect is achieved by up-regulating the mTOR pathway and increasing the expressions of glucose transporters in the jejunum of weaning offspring. Furthermore, maternal DHA supplementation increases glycogen storage within the liver and muscle, leading to improved exercise performance, and beneficially alters the structure of gut microbiome in weaning offspring. These findings lay the foundation for advocating maternal DHA supplementation during lactation as a viable strategy to foster the intestinal development of weaning offspring. However, it is important to emphasize that our study was conducted on mice, and while the results are promising, any further application to lactating women should be rigorously tested through clinical trials in human populations.

## Data availability statement

The datasets presented in this study can be found in online repositories. The names of the repository/repositories and accession number(s) can be found below: NCBI BioProject; PRJNA1102858, https://www.ncbi.nlm.nih.gov/bioproject/?term=PRJNA1102858 and ProteomeXchange; PXD052063, https://www.ebi.ac.uk/pride/archive/projects/PXD052063.

## Ethics statement

The animal study was approved by the Institutional Review Board and the Animal Care and Use Committee of Shanghai Xinhua Hospital. The study was conducted in accordance with the local legislation and institutional requirements.

## Author contributions

DL: Writing – original draft, Investigation, Methodology. DY: Writing – original draft, Methodology. GH: Investigation, Writing – original draft. JZ: Writing – original draft, Data curation. XS: Writing – review & editing, Funding acquisition. LQ: Writing – review & editing, Funding acquisition.

## Glossary

DHA: docosahexaenoic acid
LCPUFAs: long-chain polyunsaturated fatty acids
PUFAs: polyunsaturated fatty acids
GD: gestational day
PD: postpartum day
4-FDG: glucose/4-deoxy-4-fluoro-D-glucose
TC: total cholesterol
TG: total triglyceride
TP: total protein
H&E: hematoxylin and eosin
V/C: villus height to crypt depth
AUC: area under the curve
FASP: filter-aided sample preparation
DIA: data independent acquisition
GLUT2: glucose transporter 2
SGLT1: sodium glucose co-transporter 1
PEPT1: peptide transporter 1
ASCT2: alanine-serine-cysteine transporter 2
B0AT1: sodium-dependent neutral amino acid transporter 1
EAAT1: excitatory amino acid transporter 1
EAAT3: excitatory amino acid transporter 3
y + LAT1: L-type amino acid transporter 1
4F2hc: 4F2 cell-surface antigen heavy chain
CAT1: cationic amino acid transporter 1
NPC1L1: Niemann Pick C1 like 1
APOA1: apolipoprotein A-1
APOA4: apolipoprotein A-4
APOB: apolipoprotein B
FABP2: fatty acid binding protein 2
FATP4: fatty acid binding protein 4
RIPA: radioimmunoprecipitation assay
OTUs: operational taxonomic units
PCoA: principal coordinates analysis
UPGMA: unweighted pair group method with arithmetic mean
LEfSe: linear discriminant analysis coupled with effect size measurements
KEGG: Kyoto Encyclopedia of Genes and Genomes
FC: fold change
GO: gene ontology
SEM: standard error of the mean
ANOVA: analysis of variance
BP: biological process
MF: molecular function
CC: cell component
OGTT: oral glucose tolerance test
mTOR: mammalian target of rapamycin
mTORC: mTOR complex
eIF4E: eukaryotic translation initiation factor 4E
4E-BP: eIF4E-binding protein
