# Supplementary material for: Maternal docosahexaenoic acid supplementation during lactation improves exercise performance, enhances intestinal glucose absorption and modulates gut microbiota in weaning offspring mice
Source: Front Nutr. 2024 Jul 5;11:1423576. doi: 10.3389/fnut.2024.1423576 (PMC11258037; doi:10.3389/fnut.2024.1423576)
Supplement: Supplementary file 1 [file Data_Sheet_1.docx]

Supplementary Material

Supplementary Table 1. Compositions of rodent chow diet^1^ (in a food pellet form)

| Category | Composition, unit | Value |
| --- | --- | --- |
| Major components | Moisture, g/kg ≤ | 100 |
|  | Crude Protein, g/kg ≥ | 200 |
|  | Crude Fat, g/kg ≥ | 40 |
|  | Crude Fiber, g/kg ≤ | 50 |
|  | Crude Ash, g/kg ≤ | 80 |
| Minerals | Phosphorus, g/kg | 6~12 |
|  | Calcium, g/kg | 10~18 |
|  | Sodium, g/kg ≥ | 2 |
|  | Potassium, g/kg ≥ | 5 |
|  | Magnesium, g/kg ≥ | 2 |
|  | Zinc, g/kg ≥ | 0.03 |
|  | Manganese, g/kg ≥ | 0.075 |
|  | Copper, g/kg ≥ | 0.01 |
|  | Iodine, g/kg ≥ | 0.0005 |
|  | Iron, g/kg ≥ | 0.12 |
|  | Selenium, g/kg | 0.0001-0.0002 |
| Vitamins | Vitamin A, IU/g ≥ | 14 |
|  | Vitamin D, IU/g ≥ | 1.5 |
|  | Vitamin E, IU/g ≥ | 0.12 |
|  | Vitamin K (menadione), g/kg ≥ | 0.005 |
|  | Vitamin B1 (thiamin), g/kg ≥ | 0.013 |
|  | Vitamin B2 (riboflavin), g/kg ≥ | 0.012 |
|  | Niacin (nicotinic acid), g/kg ≥ | 0.06 |
|  | Vitamin B6 (pyridoxine), g/kg ≥ | 0.012 |
|  | Pantothenic Acid, g/kg ≥ | 0.024 |
|  | Vitamin B12 (cyanocobalamin), g/kg ≥ | 0.00002 |
|  | Biotin, g/kg ≥ | 0.0002 |
|  | Folate, g/kg ≥ | 0.006 |
|  | Choline, g/kg ≥ | 1.25 |
| Amino Acids | Threonine, g/kg ≥ | 8.8 |
|  | Leucine, g/kg ≥ | 17.6 |
|  | Isoleucine, g/kg ≥ | 10.3 |
|  | Valine, g/kg ≥ | 11.7 |
|  | Phenylalanine + Tyrosine, g/kg ≥ | 13 |
|  | Methionine + Cystine, g/kg ≥ | 7.8 |
|  | Lysine, g/kg ≥ | 13.2 |
|  | Histidine, g/kg ≥ | 5.5 |
|  | Arginine, g/kg ≥ | 11 |
|  | Tryptophan, g/kg ≥ | 2.5 |
| Energy | Carbohydrate (%) | 63.4 |
|  | Fat (%) | 13.8 |
|  | Protein (%) | 22.8 |

1 Grain-based rodent diet for growth and reproduction (MD17111, Jiangsu Medison Biomedical Co., Ltd.).

Supplementary Table 2. Fatty acid compositions of rodent chow diet

| Fatty acid | Value (%) |
| --- | --- |
| *Percentage of total fatty acid* |  |
| C14:0 | 2.6 |
| C16:0 | 19.2 |
| C16:1n-9 | 2.2 |
| C18:0 | 20.6 |
| C18:1n-9 | 14.6 |
| C18:2n-6 | 18.7 |
| C18:3n-3 (ALA) | 1.2 |
| C20:0 | 1.7 |
| C20:3n-6 | 0.9 |
| C20:4n-6 (AA) | 2.0 |
| C20:5n-3 (EPA) | 1.9 |
| C22:4n-6 | 0.2 |
| C22:5n-3 (DPA) | 0.4 |
| C22:6n-3 (DHA) | 1.4 |
| Total SFA | 44.1 |
| Total MUFA | 16.8 |
| n-3 PUFA | 4.9 |
| n-6 PUFA | 22.1 |

AA, arachidonic acid; ALA, α-linolenic acid; DHA, docosahexaenoic acid; DPA, docosapentaenoic acid; EPA, eicosapentaenoic acid; MUFA, monounsaturated fatty acids; n, omega; PUFA, polyunsaturated fatty acids; SFA, saturated fatty acids.

Supplementary Table 3. The primers used for qPCR in this study.

| Name | Forward | Reverse |
| --- | --- | --- |
| *GLUT2* | TCAGAAGACAAGATCACCGGA | GCTGGTGTGACTGTAAGTGGG |
| *SGLT1* | ATGCGGCTGACATCTCAGTC | ACCAAGGCGTTCCATTCAAAG |
| *β-actin* | GTACTCTGTGTGGATCGGTGG | AAAACGCAGCTCAGTAACAGTC |
| *PEPT1* | GACCACAATGGCAGTCCTGACA | ATCGCCACCAAACGCAGACACA |
| *ASCT2* | CTGCCTGTGAAGGACATCTCCT | CTCGGCATCTTGGTTCGATCCA |
| *B0AT1* | CTAGAGGAGCGGATTCCATCT | TGTTGTCCCATTTGGGCCTG |
| *EAAT1* | GTCTCGTCACAGGAATGGCG | CCTTTCCGGGGTGGATGATG |
| *EAAT3* | AAGAACCCTTTCCGCTTTG | TTGCCGAACTGGACGAGA |
| *y+LAT1* | AAGGTGTTGGCGCTGATTGCAG | AGAGTGCCAGAGCAATGTCACC |
| *4F2hc* | GAGCGTACTGAATCCCTAGTCAC | GCTGGTAGAGTCGGAGAAGATG |
| *CAT1* | CATGCCCCGAGTTATCTATGC | TTTTGGTCCTATTGTTGATTTTGG |
| *NPC1L1* | TGTCCCCGCCTATACAATGG | CCTTGGTGATAGACAGGCTACTG |
| *APOA1* | GGCAGAGACTATGTGTCCCAGT | GCTGACTAACGGTTGAACCCAG |
| *APOA4* | CAGAAGACGGATGTCACTCAGC | AGCTGTACGACAAAGGGCACCA |
| *APOB* | GCATGAGTATGCCAATGGTCTCC | CTGGTTGCCATCTGAAGCCATG |
| *CD36* | GGACATTGAGATTCTTTTCCTCTG | GCAAAGGCATTGGCTGGAAGAAC |
| *FABP2* | TCCCTACAGTCTAGCAGACGGA | CCAGAAACCTCTCGGACAGCAA |
| *FATP4* | GACTTCTCCAGCCGTTTCCACA | CAAAGGACAGGATGCGGCTATTG |

Supplementary Table 4. General body characteristics of the weaning offspring

| Characteristic | Control | L-DHA | H-DHA | *P* value |
| --- | --- | --- | --- | --- |
| Brain (mg) | 398.860 ± 2.735 | 403.120 ± 2.363 | 396.830 ± 4.437 | 0.403 |
| Heart (mg) | 60.740 ± 2.511 | 66.080 ± 2.649 | 64.050 ± 2.830 | 0.432 |
| Liver (mg) | 274.230 ± 7.572 | 295.540 ± 7.226 | 286.280 ± 6.764 | 0.130 |
| Spleen (mg) | 30.510 ± 2.804 | 36.450 ± 2.391 | 33.860 ± 2.012 | 0.239 |
| Kidney (mg) | 115.600 ± 4.273 | 124.480 ± 2.353 | 116.340 ± 2.653 | 0.113 |
| Small intestine (cm) | 22.120 ± 0.198 | 21.580 ± 0.368 | 22.300 ± 0.306 | 0.227 |
| Relative brain weight (%) | 5.453 ± 0.109 | 5.165 ± 0.152 | 5.502 ± 0.121 | 0.442 |
| Relative heart weight (%) | 0.856 ± 0.031 | 0.808 ± 0.030 | 0.883 ± 0.034 | 0.255 |
| Relative liver weight (%) | 3.867 ± 0.069 | 3.627 ± 0.099 | 3.757 ± 0.066 | 0.121 |
| Relative spleen weight (%) | 0.424 ± 0.030 | 0.429 ± 0.016 | 0.461 ± 0.030 | 0.560 |
| Relative kidney weight (%) | 1.626 ± 0.026 | 1.529 ± 0.040 | 1.563 ± 0.027 | 0.110 |

Data were expressed as the mean ± SEM (n = 10 mice per group). Statistical analysis was performed using one-way ANOVA test, with significance set at *P* < 0.05. L-DHA: 150 mg/(kg body weight · day) DHA; H-DHA: 450 mg/(kg body weight · day) DHA. DHA, docosahexaenoic acid.


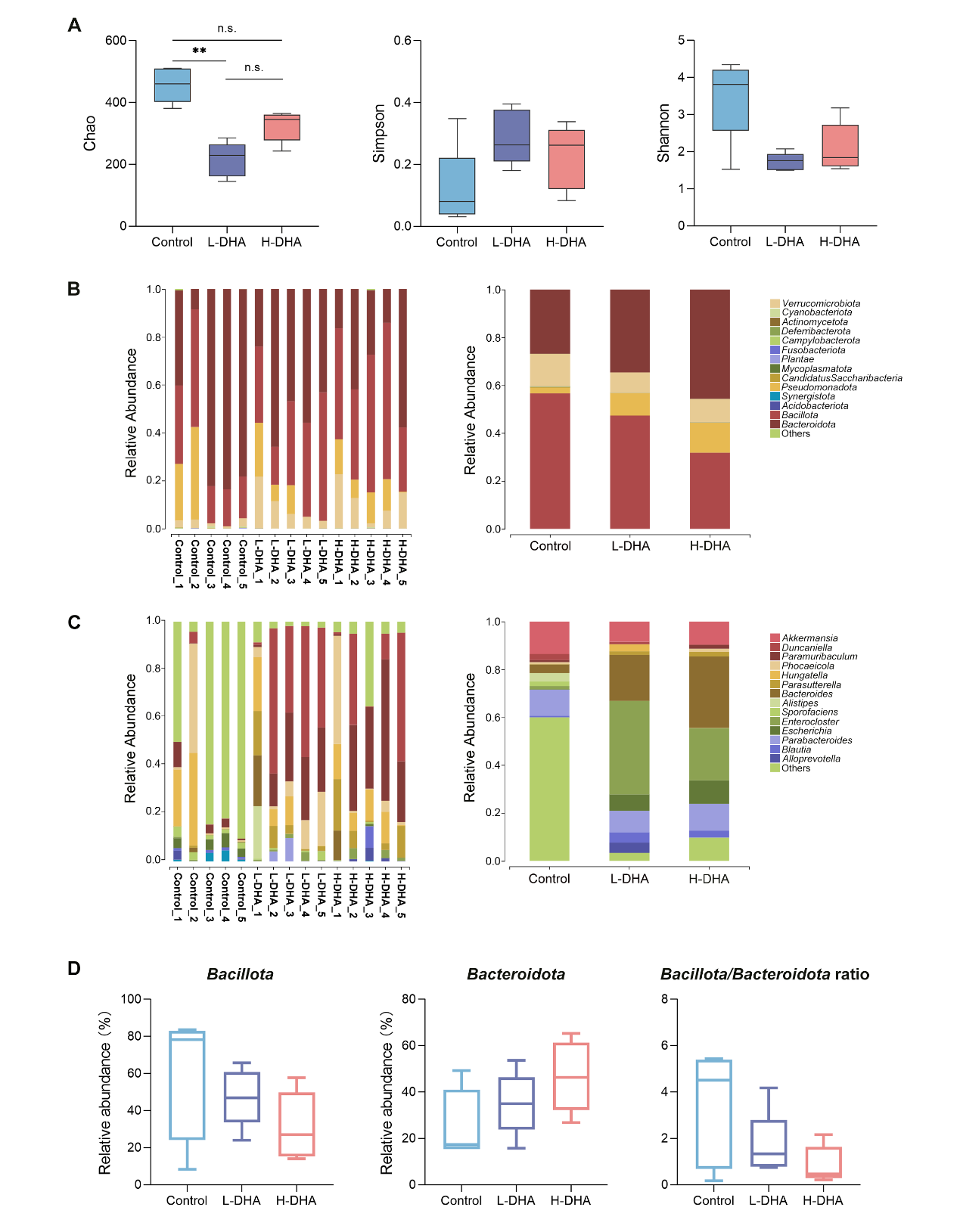


Supplementary Fig 1. (A) The *α*-diversity indices Chao, Simpson, and Shannon in different groups. (B) Relative abundance of bacterial taxa at the phylum level in each mouse and among three groups. (C) Relative abundance of bacterial taxa at the genera level in each mouse and among three groups. (D) The relative abundance of *Bacillota* and *Bacteroidetes* among three groups. Data were expressed as the mean ± SEM; n = 5 per group. Significance was set at *P* < 0.05 (***P* < 0.01). L-DHA: 150 mg/(kg body weight · day) DHA; H-DHA: 450 mg/(kg body weight · day) DHA. DHA, docosahexaenoic acid.


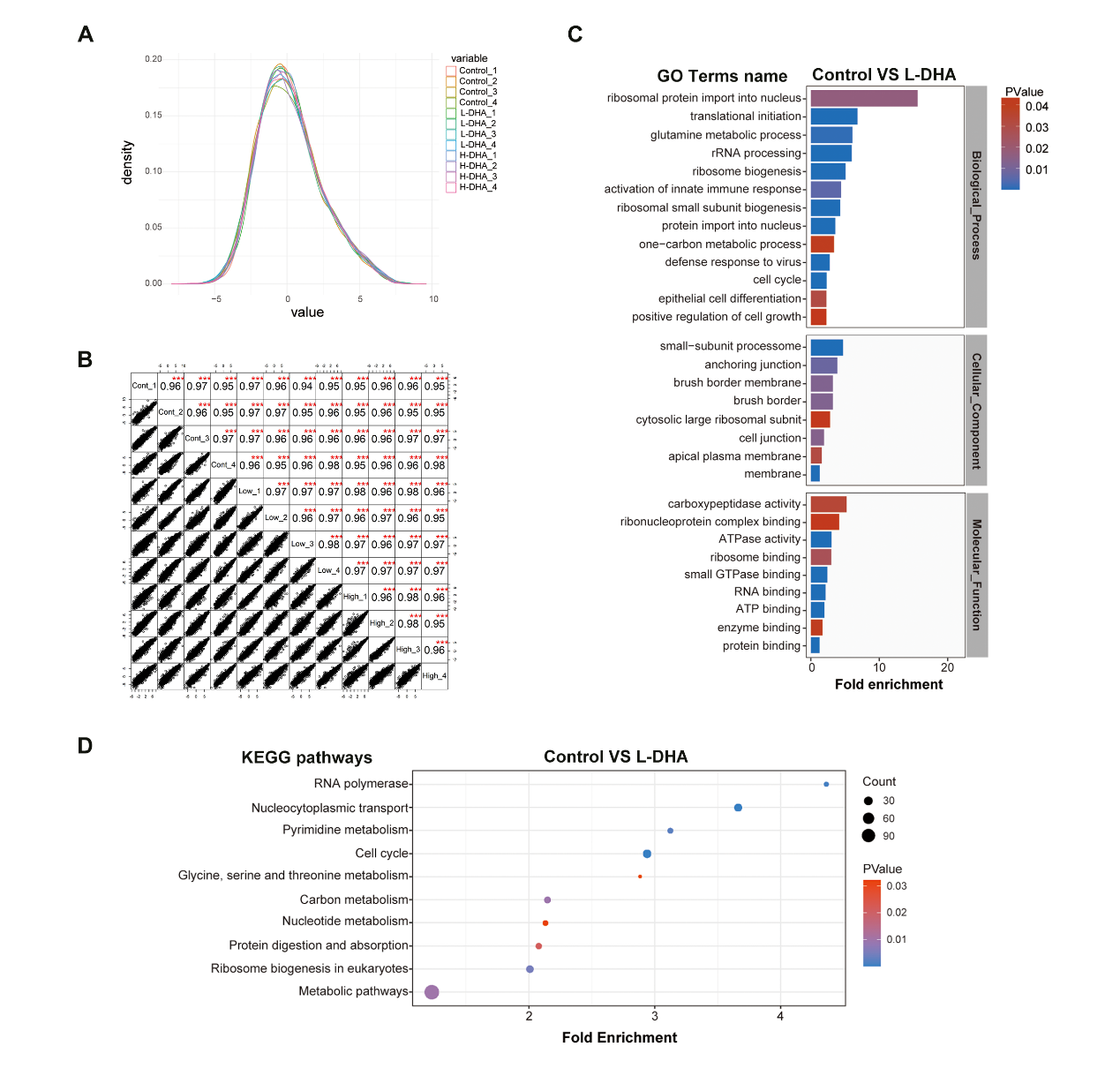


Supplementary Fig 2. Quality control of DIA-MS data and bioinformatics analysis between the control group and the L-DHA group. (A) Kernel density plot and (B) correlation analysis between samples. (C) GO analysis and (D) KEGG pathways analysis in control and L-DHA groups. n = 4 per group. L-DHA: 150 mg/(kg body weight · day) DHA. DHA, docosahexaenoic acid; DIA, data independent acquisition; GO, Gene ontology; KEGG, Kyoto Encyclopedia of Genes and Genomes.


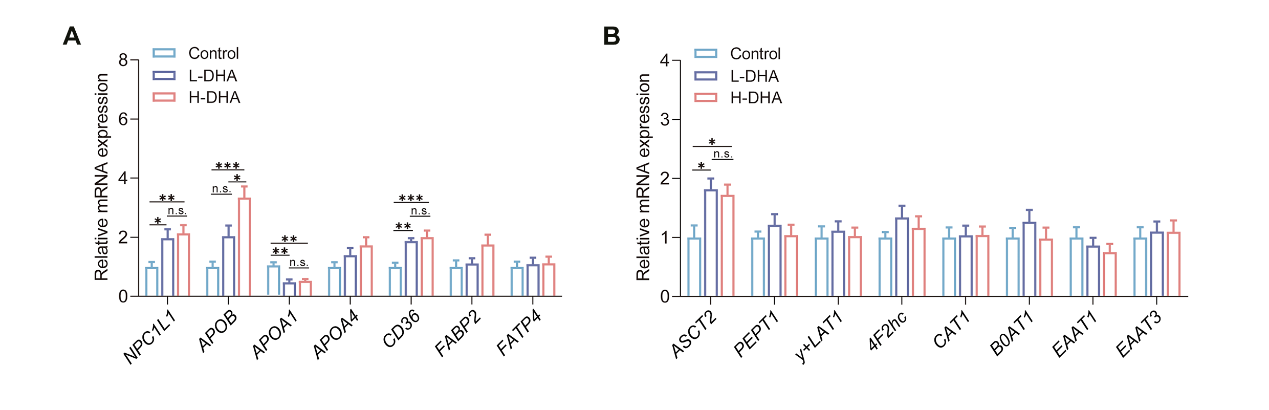


Supplementary Figure 3. Effect of maternal DHA supplementation during lactation on the jejunal mRNA expressions related to lipid and protein absorption in weaning mice. (A) Relative mRNA expressions of genes related to lipid absorption. (B) Relative mRNA expressions of genes related to protein absorption. Data were expressed as the mean ± SEM; n = 7-8 per group. Significance was set at *P* < 0.05 (**P* < 0.05, ***P* < 0.01, ****P* < 0.001). L-DHA: 150 mg/(kg body weight · day) DHA; H-DHA: 450 mg/(kg body weight · day) DHA. DHA, docosahexaenoic acid.
